# Abstracts from the Fifteenth Rambam Research Day, December 20, 2018

**DOI:** 10.5041/RMMJ.10362

**Published:** 2019-01-28

**Authors:** Shraga Blazer, Hadar Zigdon Giladi

**Affiliations:** 1 Editor-in-Chief, Rambam Maimonides Medical Journal; 2 Department of Periodontology and Implant Dentistry, Rambam Health Care Campus, Haifa, Israel; 3 Laboratory for Bone Regeneration, Rambam Health Care Campus, Haifa, Israel

We are proud to introduce you to the Fifteenth Annual Rambam Research Day, now established as a key annual event at Rambam Health Care Campus, Haifa, Israel, reflecting the diverse research activities on our campus.

Integral to Rambam’s research activities are several competitive research funding programs that Rambam administers: The Horizons (*Ofakim*) program, which provides start-up and bridge research funding for young staff physicians with strong research credentials and plans, and the Leaders (*Atidim*) program, which is directed towards research training support for our residents. The Leaders program continues to receive wide recognition and serves as an especially important example of Rambam’s dedication to medical trainees and the development of physician scientists.

Two additional competitive research grant programs were recently initiated. In 2015 the “Guardian” (*MAOF*) program for Rambam’s nursing staff was initiated. Grants of up to NIS 10,000 are awarded to support research and innovative projects of the nursing staff. The “Foundations” (*MALKAM*) research program, initiated in 2016, offers up to NIS 20,000 for resident-initiated clinical trials. Both of these programs will encourage and facilitate research initiatives from new sectors of the hospital staff and further contribute to the advancement of medical practice at Rambam. In 2017 a new program was added, benefitting our clinical laboratory researchers—the “Optimum” (*METAV*) Program.

Combined, these research programs are critical components of personal professional development for Rambam’s medical personnel. These programs enable fabrication of prototypes for medical devices and achieving preliminary results for novel ideas. In the last year, Rambam’s doctors and researchers won 29 competitive research grants, including the prestigious Israel Scientific Foundation and innovation authority grants with an estimated budget of 5 million new Israeli shekels (~1.3 million US dollars).

We would like to acknowledge the excellent collaboration and the fruitful research ties between Rambam Health Care Campus and the Technion–Israel Institute of Technology, in particular The Ruth & Bruce Rappaport Faculty of Medicine. We would also like to thank Professor Chaim (Howard) Cedar from the Hebrew University of Jerusalem as the Chair of our Scientific Advisory Board.

We are proud of the scientific abstracts that were presented at the meeting, and which compose this Supplement to *Rambam Maimonides Medical Journal*. The abstracts cover all areas of medicine and were submitted by a wide range of our staff members, including: doctors, nurses, social workers, dietitians, and researchers. They reflect the rich spectrum of clinical, applied, and basic research endeavors to develop therapeutic breakthrough from the bench to the bedside. Indeed, our primary mission of bringing health care to the community is achieved as we combine these research discoveries with excellence in teaching.

We would also like to recognize the dedicated staff of Rambam’s Research Division, including the Institutional Review Board, and the Office for Advancement of Research; together they are responsible for the coordination and oversight of all research activities at Rambam, including clinical research, applied research, competitive grant management, patent protection, institutional review board ethical approvals, and bringing forward innovative ideas to clinical practice. We value collaboration with industry, which serves as an integral part of health care progress; over the years, several Rambam-originated research projects have led to remarkable success in Israel, with practical application to health care.

We are particularly pleased to see this Supplement to *Rambam Maimonides Medical Journal*, which has now become a tradition at the *Rambam Maimonides Medical Journal*, for the awareness and promotion of research activities in Israel, particularly at Rambam. Supporting Rambam Research Day via *Rambam Maimonides Medical Journal* is another way we show commitment to our patients, via the furthering of health care facilitated by progress in science, innovation, and technology.

Rafael Beyar, M.D., D.Sc. Director, Rambam Health Care Campus; and Associate Editor, Rambam Maimonides Medical JournalHadar Zigdon Giladi, D.M.D., Ph.D. Deputy Director, Department of Periodontology and Implant Dentistry and Director, Laboratory For Bone Regeneration Rambam Health Care Campus

