# Supplementary material for: Abstracts from the Fifteenth Rambam Research Day, December 20, 2018
Source: Rambam Maimonides Med J. 2019 Jan 28;10(1):e0009. doi: 10.5041/RMMJ.10362 (PMC6363371; doi:10.5041/RMMJ.10362)
Supplement: Related texts

# Abstract: Rambam Health Care Campus Research Day Organizing Committee

**Published:** 2019-01

**Dr. Hadar Zigdon Giladi**

Director, Research Division

**Professor Shraga Blazer**

Editor-in-Chief, Rambam Maimonides Medical Journal

**Professor Benjamin Brenner**

Director, Department of Hematology and Bone Marrow Transplantation

**Professor Myriam Weyl Ben-Arush**

Director, Division of Pediatric Medicine, Ruth Rappaport Children’s Hospital, Director, Division of Pediatric Hematology-Oncology and Bone Marrow Transplantation

**Professor Doron Norman**

Chair, Division of Surgery

**Professor Zeev Weiner**

Director, Department of Obstetrics and Gynecology

**Dr. Yael Shahor**

Deputy Director, Pediatric Division, Ruth Rappaport Children’s Hospital, Attending Physician, Infectious Disease

**Assistant Professor Ron Beloosesky**

Director, Obstetrics and Gynecology Ultrasound Unit, Department of Obstetrics and Gynecology

**Dr. Gal Akiri**

Head of Research and Grants Management, Division of Research

**Mrs. Gila Hyams**

Hospital Director of Nursing and Coordinator Nurse, Trauma and Emergency Center

**Mrs. Mirit Barzilai**

Director of Nursing, Operating Rooms

**Professor Marcia C. Javitt**

Director, Department of Medical Imaging

**Assistant Professor Yeshayahu (Shai) Katz**

Director, Department of Anesthesiology

**Dr. Rostislav Novak**

Orthopedic Surgery Section

**Dr. Eyal Fruchter**

Director, Division of Mental Health

**Assistant Professor Marielle Kaplan**

Director, Department of Laboratory Medicine

**Clinical Associate Professor Amir Karban**

Director, Department of Internal Medicine C

**Dr. Aeyal Raz**

Department of Anesthesiology

**Assistant Professor Sarig Galit**

Director, Hematology Laboratory

# Abstract: Program for the Fourteenth Rambam Research Day

**Published:** 2019-01

**Thursday, December 20, 2018**

Rambam Health Care Campus, Haifa, Israel

07.00 **Meet & Greet**

07.30 **Opening Session: Greetings**

**Professor Rafael Beyar**

Director, Rambam Health Care Campus

**Professor Shimon Marom**

Dean, The Ruth & Bruce Rappaport Faculty of Medicine, Technion–Israel Institute of Technology (IIT), Haifa, Israel

**Professor Yehuda Chowers**

Director, Clinical Research Institute at Rambam (CRIR), Rambam Health Care Campus

**Honorary Lecturers**

08.00 **Professor Karen B. Avraham**

Vice Dean, The Sackler Faculty of Medicine, Tel Aviv University, Tel Aviv, Israel

Faculty Member, Department of Human Molecular Genetics and Biochemistry, Sackler Faculty of Medicine, Tel Aviv University, Tel Aviv, Israel

**Epigenomics of the Auditory System: Implications for Hearing and Deafness**

08.45 **Professor Karl Skorecki**

Dean, Azrieli Faculty of Medicine, Bar-Ilan University, Safed, Israel

**Diversity Drives Discovery**

09.40 **Professor Rafael Beyar**

Director, Rambam Health Care Campus, Haifa, Israel

Women’s Division/Dr. Phillip and Sara Gotlieb, Professor of Medicine and Biomedical Engineering, The Ruth & Bruce Rappaport Faculty of Medicine, Technion–Israel Institute of Technology, Haifa, Israel

**Innovations in Medicine: From the Bench to the Bedside**

10.15 **Best Rambam Research Day Oral Presentations**

11:30 **Announcement of Internal Rambam Research Grant Recipients**

# Abstract: Epigenomics of the Auditory System: Implications for Hearing and Deafness

**Published:** 2019-01

**Authors:** Karen B. Avraham

**Affiliations:** Department of Human Molecular Genetics & Biochemistry, Sackler Faculty of Medicine and Sagol School of Neuroscience, Tel Aviv University, Tel Aviv, Israel

Given that hearing loss affects hundreds of millions of people worldwide, the challenge in auditory science is to determine how a pathogenic variant in a gene or regulatory element can cause the entire hearing system to fail. Our team is asking the following questions: (1) What are the genes that lead to hearing loss, and how are they involved in normal function of the inner ear? (2) How does regulation of gene expression govern the pathways that determine inner ear function, and how do alterations in regulation, on a genetic and epigenetic level, contribute to the pathology of deafness? To answer the first question, we have studied Israeli and Palestinian families with inherited hearing loss to identify genetic pathogenic variants, as well as decipher the related mechanisms leading to deafness. We incorporate the use of mouse models for human deafness and, most recently, have used CRISPR-Cas9 genome editing to create these models. To answer the second question, we are exploring a genome-wide view of the regulatory elements playing a role at different steps of maturation and functionality of the inner ear. We investigate multiple levels of epigenetic regulation of cell circuitry in the mouse inner ear sensory epithelium and hair cells, including the transcriptome, chromatin structure, histone modification, and DNA methylation, using advances in high-throughput sequencing coupled with technologies such as ATAC-Seq, ChIP-Seq, and MethylC-Seq. The above studies are providing a thorough and detailed understanding of regulatory pathways that control the auditory system, toward development of biological therapies for hearing impairment.

# Abstract: Diversity Drives Discovery

**Published:** 2019-01

**Authors:** Karl Skorecki

**Affiliations:** Dean, Azrieli Faculty of Medicine, Bar-Ilan University, Safed, Israel

Global human diversity can be partitioned into genomic, post-genomic, biological (e.g. epigenetic, microbiomic), and cultural components. Genomic diversity has arisen from mutational and recombination processes, acted upon by either neutral demographic or selective evolutionary forces. The genomic architecture of common non-Mendelian heritable diseases (e.g. diabetes, asthma, atherosclerotic cardiovascular disease, young adulthood cancer, hypertension, kidney disease, neurodegenerative disease, and many others) governs the choice of an optimal research discovery approach. For example, the predominant contribution of one or a few rare disease risk alleles in known genes would favor full sequencing approaches applied to large cohorts, or multiplex high-risk families. This approach can yield disease risk causative genes and mutations, which can serve as therapeutic targets. The BRCA loci mutations conferring risk for solid tumors at multiple sites is a prominent example with immediate clinical implications. Risk for abdominal aortic aneurysm is another example. In contrast, the infinitesimal additive contribution of hundreds or thousands of genetic variants to major disease risk requires very large sample sets which can be processed in microarrays of known variants. This can yield a polygenic risk score, but not a disease-causative target. Risk for myocardial infarction and for prostate cancer are well-described examples. Since genetic variation can also be partitioned by continental ancestries, it is not surprising to find that certain population health disparities have a genetic basis. The strong association of high-frequency allelic variants at the human apolipoprotein L1 gene with greatly increased risk for chronic kidney disease and its complications in populations of sub-Saharan African descent provides a powerful example with immediate patient management implications and drug discovery opportunities. This example can be used as a template for identifying targets and developing novel therapies for many forms of widespread non-communicable disease.

# Abstract: Innovations in Medicine: From the Bench to the Bedside

**Published:** 2019-01

**Authors:** Rafael Beyar

**Affiliations:** Director, Rambam Health Care Campus, Haifa, Israel; and The Ruth & Bruce Rappaport Faculty of Medicine, Technion–Israel Institute of Technology, Haifa, Israel

Medicine is advancing through technology, with collaborations between clinicians, scientists, and engineers being essential elements in the process of innovation. Innovation starts from an unmet clinical need in combination with technological advancement. The innovation can be led by physician practitioners who see the unmet need, by individual entrepreneurs, by the engineers and scientists in academia, or by industry; however, to be successful, innovation needs collaboration between all these partners.

If we focus on medical devices, innovations are composed of endless cycles of processes starting from the unmet need, through to a patent application and securing the future value of the idea, on to prototype design, animal experiments, then first-in-human experiments, and finally pivotal studies for regulatory approval. For an innovation to have a clinical impact, the correct reimbursement model that allows its use by the general public is mandatory and must already be considered early in the process of development.

Innovations can be classified as disruptive or expanding. Disruptive innovations change the established behaviors of the field, whereas an expanding innovation simply continues and improves upon current technologies. Examples of disruptive innovations in the field of cardiology are balloon angioplasty and stents, both of which completely changed the way patients are treated today.

Programs to enhance innovations in medicine are extremely important. Israel is recognized worldwide for its medical innovations. There is a strong culture of entrepreneurs in Israel, which is dependent upon academic hospitals, universities, industry, the government, and private support. The incubator program in Israel has been an important tool for enhancing innovations in medicine, which typically take much more time and effort than other innovative processes due to the regulatory processes that are required to gain approval. Rambam Health Care Campus exemplifies such collaborations: the hospital is affiliated with the Technion–Israel Institute of Technology; it has its own technology transfer company, Rambam MedTech; and the hospital has partnered with industry to create the digital and medical device incubator, MindUp. The establishment of an interactive collaborative structure thus enhances medical innovation and helps transfer solutions to real clinical needs from bench to bedside.

# Abstract #012. Seven versus 14 Days of Antibiotic Treatment of Gram-negative Bacteremia—a Non-inferiority Randomized Controlled Trial

**Published:** 2019-01

**Authors:** Mical Paul, Fidi Koppel, Roni Bitterman, Ami Neuberger, Nesrin Ghanem-Zoubi, Anat Stern, Yaakov Dickstein, Hiba Zbu-Zayyad, Cristina Mussini, Leonard Leibovici, Dafna Yahav

**Affiliations:** 1 Infectious Diseases Institute, Rambam Health Care Campus, Haifa, Israel; 2 The Ruth & Bruce Rappaport Faculty of Medicine, Technion–Israel Institute of Technology, Haifa, Israel; 3 Department of Infectious Diseases, Modena University Hospital, Modena, Italy; 4 Modena University, Modena, Italy; 5 Medicine E, Rabin Medical Center, Beilinson Hospital, Petah Tikva, Israel; 6 Sackler Faculty of Medicine, Tel Aviv University, Ramat Aviv, Israel; 7 Unit of Infectious Diseases, Rabin Medical Center, Beilinson Hospital, Petah Tikva, Israel

## Background

Gram-negative bacteremia is a major cause of morbidity and mortality in hospitalized patients. Data to guide the duration of antibiotics are limited.

## Study Aims

The aim of this study was to assess the safety and efficacy of short-course (7 days) antibiotic treatment for Gram-negative bacteremia.

## Materials & Methods

Open-label, non-inferiority randomized controlled trial conducted in three medical centers in Israel and Italy. Inpatients with Gram-negative bacteremia, afebrile and hemodynamically stable for at least 48 hours, were randomized at a 1:1 ratio to receive 7 vs. 14 days of *in vitro* covering antibiotics. Randomization was computer-generated and concealed using sealed opaque envelopes. Patients with uncontrolled focus of infection were excluded. The primary outcome was a composite of: all-cause mortality; relapse of bacteremia, suppurative or distant complications; and re-admission or extended hospitalization (>14 days), all assessed at 90 days. The non-inferiority margin was 10%. Analyses are reported for the intention-to-treat population.

## Results

We included 604 patients between January 2013 and August 2017. Source of infection was urinary in 411/604 (68%) patients; causative pathogens were mainly Enterobacteriaceae (543/604, 90%). A difference of 7 days in the median duration of antibiotics between study arms was achieved.

The primary outcome occurred in 140/306 (45.8%) patients in the 7 days group vs. 144/298 (48.3%) in the 14 days group (risk difference [RD] −2.6%, 95% confidence interval [CI] 10.5% to 5.3%). Non-inferiority was demonstrated also for 90-day all-cause mortality, with 36 (11.8%) deaths in the short duration group and 32 (10.7%) deaths in the long duration group (RD 1.0%, 95% CI −4% to 6.1%). Time to return to baseline activity within 90 days was significantly shorter in the short duration arm (median 2 weeks [IQR 0–8.3] vs. median 3 weeks [IQR 1–12 weeks]). Adverse events, including acute kidney injury and diarrhea, were without significant difference between study arms (14/306, 4.6% vs. 12/298, 4%, RD 0.5, 95% CI −2.7 to 3.8; and 49/306, 16% vs. 54/298, 18.1%, RD −2.1, 95% CI −8.1 to 3.9, respectively).

## Conclusions

In patients hospitalized with Gram-negative bacteremia who achieved clinical stability before day 7, an antibiotic course of 7 days was non-inferior to 14 days. Reducing antibiotic treatment for bacteremia to 7 days is an important antibiotic stewardship intervention.

# Abstract #017. Unraveling the Diverse Interactions of Leukemic Cells with the Bone Marrow Microenvironment

**Published:** 2019-01

**Authors:** Shlomit Yehudai-Reshef, Rawan Sabah, Anna Fridman-Dror, Tal Gabay, Shira Turgeman, Tsila Zuckerman

**Affiliations:** 1 Hematology Research Center, The Clinical Research Institute at Rambam, Rambam Health Care Campus, Haifa, Israel; 2 Hematology and Bone Marrow Transplantation Institute, Rambam Health Care Campus, Haifa, Israel

## Background

Acute myeloid leukemia (AML) is caused by genetic aberrations in hematopoietic stem cells (HSCs), leading to the accumulation of immature cells (blasts) in the bone marrow (BM) and peripheral blood due to differentiation inhibition of early progenitors. Despite intense research and new treatments, patient survival has not significantly improved in recent decades. Hence, better understanding of the disease pathogenesis is required in order to develop novel strategies for AML management. Most AML studies have focused on the genetic evolution of the disease with the premise that somatic genetic alterations (mutations) found in the pre-leukemic and leukemic cells drive leukemogenesis. However, recent studies using next-generation sequencing to explore the genetic landscape of AML in large patient cohorts have demonstrated that approximately 10%–40% of AML patients exhibit the normal genotype, i.e. their leukemic blasts do not harbor detectable mutations that can explain their disease. This suggests potential involvement of other mechanisms in disease initiation.

The BM microenvironment (ME) is known to indirectly contribute to AML pathogenesis, resistance to chemotherapy, and recurrence. However, there is no evidence that BM mesenchymal stromal cells (MSCs) are responsible for AML initiation and propagation.

## Study Aims

The aim of this study was to unravel the cross-talk of leukemic cells with the BM ME and elucidate the role of bone niche in leukemogenesis. We hypothesized that a patient’s BM niche is involved in leukemogenesis and that targeting the pathological BM-derived cells, rather than AML cells themselves, may prevent AML initiation/propagation and relapse.

## Materials & Methods

We analyzed AML-derived BM MSCs compared to BM MSCs obtained from healthy subjects. Additionally, we explored morphological differences between AML MSCs and healthy MSCs, as well as cytokine secretion profiles in various cultures.

We developed an *ex vivo* system of the patient’s own stroma (POS), which simulates the *in vivo* BM ME. The POS derived at different disease stages (diagnosis, remission, and relapse) was used to study properties of BM MSCs and their cross-talk with AML cells.

## Results

Compared to MSCs obtained from healthy volunteers, AML MSCs demonstrated larger cell areas (154.34 μm^2^ in active disease and 187.97 μm^2^ in remission vs. 47 μm^2^ in healthy MSCs, *P*=0.001 and *P*=0.024, respectively), greater heterogeneity in cell shape, and a slower growth rate. Additionally, AML cells showed superior proliferation in direct contact with POS compared to MSCs derived from healthy volunteers (*P*<0.0001). Likewise, the expression level of osteopontin, a cytokine known to be a mediator in the interaction of POS and AML cells, was higher in POS from active disease and remission (2.5±16.6 arbitrary units (AU) and 2.1±97.9 AU, respectively) than in healthy MSCs (0.5±0.4 AU; *P*=0.022, *P*=NS). Remarkably, remission-derived MSCs, which are considered healthy, displayed features similar to those of MSC obtained in active disease. Exome sequencing of AML MSCs revealed the existence of three somatic mutations associated with various MSCs differentiation pathways (AHNAK2, FoxD4L1, ATP5A1) in three out of five AML patients.

## Conclusions

Overall, this study brings new insights to the contribution of BM ME to leukemogenesis. This work may pave the way towards novel personalized AML treatment targeting the tumor ME.

# Abstract #024. Use of Intraoperative Electrocochleography for Hearing Preservation and Localization in Cochlear Implant Surgery

**Published:** 2019-01

**Authors:** Mauricio Cohen-Vaizer, Eran Fridman, Shifaa Dakhlallah, Moria Federman

**Affiliations:** Department of Otolaryngology, Head and Neck Surgery, Rambam Health Care Campus, Haifa, Israel

## Background

Hearing and structural preservation in cochlear implantation (CI) are associated with the ability to use hybrid devices (acoustic and electrical input). Intraoperative electrocochleography (ECochG) has been proposed as a means for providing real-time feedback to the surgeon.

## Study Aims

This study evaluated the usefulness of intraoperative monitoring during cochlear implantation.

## Materials & Methods

This was a pilot, controlled, observational study. Eleven CI patients underwent surgery with the aid of intraoperative ECochG and were compared with a similar group of patients who underwent surgery without the aid of ECochG.

## Results

Differences were observed in insertion time and strategy, follow-up impedances, and intraoperative and postoperative hearing preservation. Additionally, ECochG provided valuable information about the position of the electrode lead.

## Conclusions

Intraoperative ECochG significantly affects the results of CI surgery.

# Abstract #027. Combination of Heparanase Inhibitors and Aspirin Dramatically Ameliorates Acute Pancreatitis in an Animal Model

**Published:** 2019-01

**Authors:** Iyad Khamaysi, Dalit Ben Hemo, Udayan Bhattacharya, Safa Kinaneh, Neta Ilan, Israel Vlodavsky, Zaid Abassi

**Affiliations:** 1 Department of Gastroenterology and Hepatology Unit, Rambam Health Care Campus, Haifa, Israel; 2 The Ruth & Bruce Rappaport Faculty of Medicine, Technion–Israel Institute of Technology, Haifa, Israel

## Background

Acute pancreatitis (AP) is one of the most common diseases in gastroenterology. Its pathophysiology, however, is still not fully recognized. Therefore, no specific or effective treatment has been developed. Previously, we used an animal model to demonstrate the important role that heparanase (Hpa) plays in the pathogenesis of AP, and that Hpa inhibitors significantly reduced the severity of the disease. Most recently, aspirin has also been shown to possess an inhibitory effect against Hpa activity both *in vivo* and *in vitro*. We therefore proposed that, by combing specific Hpa inhibitors with aspirin, we could ameliorate AP more efficiently than each drug alone.

## Study Aims

This study assessed whether combining either PG545 (pixatimod) or SST0001 (roneparstat), two specific Hpa inhibitors, with aspirin exerts a superior pancreato-protective effect in cerulein-induced AP in mice, compared to either compound alone.

## Materials & Methods

Six groups each of wild-type (WT) BALB/c mice and heparanase-overexpressing transgenic animals (Hpa-TG) (*n*=7–8/group) were treated every hour for 5 hours, as follows: control, intraperitoneal (i.p.) normal saline alone; cerulean alone, 50 mg/kg i.p.; cerulean plus pixatimod (0.4 mg/mouse) or roneparstat (2 mg/mouse), i.p.; and cerulean plus pixatimod or roneparstat with aspirin (250 mg/kg subcutaneously). Twenty-four hours from the induction of acute pancreatitis, the animals were sacrificed. The severity of AP and architectural structure changes were evaluated by serum amylase and lipase levels, inflammatory cytokines, pancreatic edema index (determined by the ratio of pancreas weight to body weight), pancreatic histological alterations (determined by light microscopy and H&E staining), inflammatory response (determined by immune-histopathological analysis), and autophagy response (determined by electron microscopy and immunohistochemistry staining).

## Results

Cerulein-induced AP in WT mice was characterized by significant rises in the serum levels of amylase (×2.5) and lipase (×4). These increases were associated with enhancement of pancreatic edema index, tissue inflammation, and autophagy response. All these AP typical responses were profoundly exaggerated in Hpa-TG mice, as was evident by 6- and 8-fold increases in amylase and lipase levels, respectively. Noteworthy, pretreatment with pixatimod, roneparstat, or aspirin attenuated pancreatic inflammatory response, autophagy, and the elevation of amylase and lipase serum levels in WT and Hpa-Tg mice. It should be emphasized that a combination of pixatimod or roneparstat with aspirin completely abolished AP at the biochemical, inflammatory, and histological levels in both subgroups of animals.

## Conclusions

Administration of either Hpa inhibitor or aspirin exerts a protective effect against cerulein-induced AP. Interestingly, combinations of aspirin and Hpa inhibitors completely ameliorated AP, providing the rational basis for a novel therapeutic approach to this disease.

# Abstract #054. Using Advanced hiPSC Models to Study Complex Cardiac Arrhythmias and Treatments of the Short QT Syndrome

**Published:** 2019-01

**Authors:** Rami Shinnawi, Naim Shaheen, Irit Huber, Assad Shiti, Gil Arbel, Amira Gepstein, Anke Tijsen, Marin Borggrefe, Lior Gepstein

**Affiliations:** 1 Department of Cardiology, Rambam Health Care Campus, Haifa, Israel; 2 The Ruth & Bruce Rappaport Faculty of Medicine, Technion–Israel Institute of Technology, Haifa, Israel; 3 First Department of Medicine, Faculty of Medicine, University Medical Center Mannheim (UMM), University of Heidelberg, Mannheim, Germany

## Background

Congenital short QT syndrome (SQTS) is an inherited arrhythmogenic syndrome caused by abnormal ion channel function, leading to abbreviated action-potential duration (APD) and life-threatening arrhythmias, resulting in sudden cardiac death.

## Study Aims

There were five study aims: (1) To develop a patient-specific human induced pluripotent stem cell (hiPSC) disease model of congenital SQTS at the cellular level. (2) To establish an isogenic control line using gene-editing technology. (3) To develop a patient-specific two-dimensional hiPSC cardiac sheet (hiPSC-CS) model of the SQTS. (4) To study the electrical properties of the generated hiPSC-CSs. (5) To assess the hiPSC-CS response to drug interventions.

## Materials & Methods

Patient-specific hiPSC lines were generated from both a symptomatic SQTS patient carrying missense mutation (N588K) in the hERG gene, leading to gain-of-function in the rapidly activating delayed rectifier potassium current (I_Kr_), and from a healthy subject. A CRISPR-Cas9 system was used to create an isogenic control line. All hiPSC lines were directly coaxed to differentiate into hiPSC-derived cardiomyocytes (hiPSC-CMs). Action-potentials and I_Kr_ current were measured using whole-cell recordings; hiPSC-CMs were seeded as circular 5 mm-diameter homogeneous hiPSC-CS, loaded with a voltage-sensitive dye (FluoVolt), and subjected to detailed optical mapping studies.

## Results

At the single-cell level, the SQTS-hiPSC-CMs recapitulated the disease phenotype showing abbreviated APD, shortened refractory period, and higher I_Kr_ end-pulse currents (due to attenuated inactivation), compared to both healthy and isogenic controls. At the tissue level, optical mapping studies of the SQTS-hiPSC-CSs revealed abbreviated APD, yet a similar conduction velocity compared to both controls. Moreover, SQTS-hiPSC-CSs demonstrated impaired APD rate adaptation, in conjunction with the characteristic impaired rate adaptation of QT interval in SQTS patients.

Applying an arrhythmia induction protocol on SQTS-hiPSC-CSs resulted in higher inducibility of sustained functional reentries characterized by higher rotor frequency, higher rotor curvature, and decreased meandering (resulting in higher rotor stability) compared to both controls. Furthermore, 1 μM quinidine (the drug of choice) normalized APD and prevented sustained reentry inducibility in SQTS-hiPSC-CSs. However, 5 μM sotalol did not significantly prolong APD nor affect reentry inducibility, along with its lack of effectiveness in SQTS1 patients.

## Conclusions

A novel hiPSC-based model of the SQTS was established at the single-cell and tissue levels. Gene-editing technology allowed establishing isogenic control and rescuing the diseased phenotype. This model recapitulated the disease phenotype and provided novel mechanistic insights into arrhythmogenicity in SQTS. Furthermore, the differential response to quinidine and sotalol reflected the patient response for those drugs, demonstrating the potential of the model for drug screening.

# Abstract #001. Machine Learning Approach to Fetal Weight Estimation

**Published:** 2019-01

**Authors:** Ido Solt, Or Caspi, Ron Beloosesky, Zeev Weiner, Eyal Avdor

**Affiliations:** 1 Department of Obstetrics and Gynecology, Rambam Health Care Campus, Haifa, Israel; 2 The Ruth & Bruce Rappaport Faculty of Medicine, Technion–Israel Institute of Technology, Haifa, Israel; 3 Medical Data Insights (MDI), Tel-Aviv, Israel

## Background

Fetal weight estimation (FWE) is important for ensuring the safety of both the fetus and mother. Current estimation methods are based on sonography binned into quantiles and then compared to reference growth curves. It is well established that this method is inaccurate, and there is a debate regarding the best approach.

## Study Aims

We propose a novel FWE approach that uses all collected multidimensional data rather than a predetermined quantile, in order to create a standardized model.

## Materials & Methods

We performed a retrospective study on a total of 6,972 patient records in a large referral center from January 2011 to December 2016. A trained physician performed an ultrasound estimation of fetal weight on every parturient presented to the delivery room. The fetal weight estimations were linked with postpartum data. Information included: date and time of admission, demographics, gestational age, ultrasound measurements (biparietal diameter [BPD], femoral length [FL], abdominal circumference [AC], head circumference [HC]), and newborn sex. Since real-life data were used, exclusion criteria included examinations with missing data or illogical values. After normalizing the data, we applied nine supervised learning algorithms to create a new FWE formula.

We then applied the K-Means algorithm on the data to create clusters based on the measurements, and then created five different estimators for the different clusters.

We used a training dataset with 4,880 entries, a 10-fold cross-validation set, and a test dataset with 2,092 entries.

To evaluate the quality of the models, we examined the mean absolute error (MAE) with respect to the actual birthweight (grams) and compared them using three Hadlock formulas. We also calculated the *R2* value for each model.

## Results

When using machine learning algorithms on the entire dataset to create one equation for FWE, the clustered estimator achieved the best results. In a comparison of Hadlock formulas to machine learning algorithms in predicting birthweight, the mean absolute errors in grams (lower is better) were: AdaBoost Regressor, 231.8; Hadlock 1 (AC, FL), 229.9; Hadlock 2 (HC, AC, FL), 226.5; RANSAC, 224.0; Hadlock 3 (HC, BPD, AC, FL), 17.3; Linear Regressor, 213.4; Support Vector Regressor, 213.3; Pipeline Regressor, 212.6; and Clustered Regressor, 210.9.

The *R2* values for the *different* models were: 0.7, 0.695, 0.693, 0.672, 0.645, 0.641, 0.638, 0.613, and 0.256, respectively.

## Conclusions

A machine learning algorithm approach to FWE creates models that reach a higher level of accuracy compared to the currently used methods.

# Abstract #003. Intestinal Dysbiosis in Carbapenem-resistant Enterobacteriaceae Carriers

**Published:** 2019-01

**Authors:** Hilla Korach Rechtman, Maysaa Hreish, Carmit Fried, Shiran Gerassy-Vainberg, Zaher S. Azzam, Yechezkel Kashi, Gidon Berger

**Affiliations:** 1 Faculty of Biotechnology and Food Engineering, Technion–Israel Institute of Technology, Haifa, Israel; 2 The Ruth & Bruce Rappaport Faculty of Medicine, Technion–Israel Institute of Technology, Haifa, Israel; 3 Department of Internal Medicine B, Rambam Health Care Campus, Haifa, Israel

## Background

Carbapenem-resistant Enterobacteriaceae (CRE) are drug-resistant pathogens with a rapidly increasing infection incidence in a variety of clinical settings. They are associated with increased morbidity and mortality. Infections caused by CRE have been associated with increased cost and length of hospitalization as well as frequent treatment failures and death. In order to establish itself in the gastrointestinal tract (GIT) and complete colonization, CRE must successfully compete with many co-occurring microorganisms. It was shown that microbiota undergo dramatic changes in different diseases, therefore we hypothesize that imbalanced GIT-associated microbiota enables CRE colonization.

## Study Aims

The aim of this study was to determine the composition of the GIT microbiota in CRE-colonized patients compared to non-carriers, and to associate CRE-carriage with microbial population structure and function.

## Materials & Methods

In order to characterize the structure of GIT microbiota we utilized high-throughput 16S rRNA gene sequencing. DNA was extracted from fecal samples collected from hospitalized CRE-carriers (*n*=40) and from two control groups: hospitalized non-carriers (*n*=22) and healthy adults (*n*=15).

## Results

The microbiota diversity and composition in CRE-colonized patients were found to differ from those of the participants in the two control groups. These CRE-colonized patients displayed lower phylogenetic diversity and dysbiotic microbiota, enriched with Enterobacteriaceae family bacteria, especially *Enterobacter*, *Erwinia*, *Pantoea*, and *Klebsiella* genera. Concurrent with the bloom in Enterobacteriaceae family, a depletion of anaerobic commensals (Rikenellaceae family, *Barnesiella* and *Ornithobacterium* genera [phylum Bacteroides], *Faecalibacterium*, *Ruminococcus* spp., and *Coprococcus* [phylum Firmicutes]) was observed.

Concomitant with the microbiota alteration, we found a higher prevalence of bacteremia in the CRE carriers, which can be explained by several predicted microbial metabolic pathway alterations. These include enrichment in xenobiotics biodegradation and metabolism in CRE-carriers, which are known to promote Enterobacteriaceae family growth and virulence. Other pathways that differed between CRE-carriers and the two control groups include pathways that correlate with repression of the immune system response to pathogens and pathways that cause damage to the intestinal epithelial barrier and function, which allow bacterial translocation to the blood stream. Several clinical factors that might induce change in the microbiota were examined, with no significant difference noted between the three groups, except for pathogen carriage.

## Conclusions

Our study demonstrated that CRE-colonized patients have dysbiotic gut microbiota in terms of diversity and community membership, as well as high rates of systemic infection.

# Abstract #007. Melanoma Epidemiology in Northern Israel

**Published:** 2019-01

**Authors:** Ori Samuel Duek, Yeela Ben Naftali, Yehuda Ullmann

**Affiliations:** Department of Plastic Surgery, Rambam Health Care Campus, Haifa, Israel

## Background

The population of Israel is divided into various ethnic groups: Jewish groups (fair-skinned Ashkenazic Jews [AJ] who immigrated from Europe; darker-skinned Sephardic Jews [SJ] who immigrated from the Orient and Africa) and darker-skinned Arabs. Among the Jewish groups were also Israeli-born descendants of the AJ and SJ groups as well as a large number of relatively new immigrants from the last three decades (mostly from the former Soviet Union). The biologic behavior of malignant melanoma (MM) in these different groups, and the effect of immigration to a sunny country on MM development, have not yet been investigated.

## Study Aims

This study aimed to compare MM histologic findings and disease spread in the different epidemiologic groups in northern Israel, and to evaluate the effect of immigration on MM occurrence.

## Materials & Methods

This was a retrospective review of the medical records for all melanoma patients admitted to the Department of Plastic Surgery or who visited the plastic surgery clinic of a single tertiary center in northern Israel. In total 130 patients undergoing melanoma excision during a 1-year period (April 2016–April 2017) were identified and studied. Patient demographics (e.g. age, sex, ethnic group), histologic findings, lymph node invasion and distant metastases, adjuvant therapy, and survival parameters were analyzed.

## Results

Analyses revealed that immigrants from Europe or North America were at increased risk for melanoma compared with immigrants from African or Asian countries. The increased risk of melanoma was also statistically significant among a large subset of European immigrants from the former Soviet Union, who accounted for 32% of all melanoma patients in the study group, compared to 9% of the total population in Israel (*P*<0.05). Most of the melanoma tumors in this population of immigrants were found in upper and lower extremities (60%), with diagnoses at a younger age compared to other European immigrants (*P*<0.05), with a trend towards a more advanced disease than the melanoma found in other patients.

## Conclusions

The study findings indicate that immigrants from the former Soviet Union are at an increased risk of developing melanoma as compared to the rest of the population. Raising awareness in this population group regarding preventative measures and the importance of early diagnosis may reduce the morbidity and mortality caused by the disease. Further research is needed to determine whether routine screening tests should be provided for this population.

# Abstract #008. Merkel Cell Carcinoma—A Retrospective Study

**Published:** 2019-01

**Authors:** Ori Samuel Duek, Yeela Ben Naftali, Yehuda Ullmann

**Affiliations:** Department of Plastic Surgery, Rambam Health Care Campus, Haifa, Israel

## Background

Merkel cell carcinoma (MCC) is a rare aggressive neuroectodermal skin cancer, with frequent recurrences and a high mortality rate. Risk factors for MCC include ultraviolet light exposure, advanced age, and immunosuppression.

## Study Aims

The purpose of this study was to describe a cohort of MCC patients admitted to Rambam Health Care Campus, and to compare their characteristics and disease behavior to the literature.

## Materials & Methods

This was a retrospective review of medical records for all MCC patients admitted to the plastic and reconstructive surgery department of a single tertiary referral center in northern Israel. In total 17 patients undergoing MCC excision during a 5-year period (July 2011–July 2016) were identified and studied. Patient demographics, medical history, histologic findings, lymph node invasion and distant metastases, adjuvant therapy, recurrence, and survival parameters were analyzed.

## Results

The patients in the study group are elders, mostly men of European origin. Immune suppression was described as a risk factor only for a minority of the patients. The link between melanoma and MCC is known, and, indeed, about a quarter of the patients also had melanoma (twice as prevalent as in the general population). In contrast, diabetes is not described as a risk factor for MCC, but occurred in about 50% of the cases, more than five times as prevalent as in the general population. The distribution of the anatomical location of the primary tumor was consistent with the literature. In 50% of the cases, the primary tumor was in the head–neck area, 28% in the upper extremities, 11% in the lower extremities, and 11% in the trunk. Metastases could be found anywhere in the body, as described in the literature, and was seen in about 20% of cases at the time of diagnosis. In one-third of the patients, lymph nodes were involved during surgery, and 50% recurrence was observed with approximately 40% of patients in the study group within 5 years.

## Conclusions

The findings of this study may imply, for the first time, a relationship between diabetes and MCC, a connection that may explain the rise in the incidence of the disease in the last decades. In addition, a literature review was carried out and therapeutic principles were defined for a metastatic disease, principles that place the innovative immunotherapy therapies in the center of the treatment regimen, in the hope of improving the prognosis of patients suffering from this aggressive disease.

# Abstract #010. Plastic Surgery in the Social Media

**Published:** 2019-01

**Authors:** Yeela Ben Naftali, Ori Samuel Duek, Yehuda Ullman

**Affiliations:** Department of Plastic Surgery, Rambam Health Care Campus, Haifa, Israel

## Background

The use of social media is growing tremendously along with its impact on the practice of plastic surgery, for better or for worse. Patients are increasingly using social media to obtain information about either the procedure or the surgeon.

## Study Aims

The aims of this study were to examine social media postings relating to plastic surgery, and to analyze successful online communication methods of the plastic surgeons with the public.

## Materials & Methods

A prospective analysis of three popular, global social media networks was performed, using the key phrases “plastic surgery” and “#plastic_surgery.” Three hundred posts relating to plastic surgery and published on Instagram, YouTube, or Facebook in November 2017 were assessed based on the following parameters: author identity, subject, “social media currency” (likes, shares, comments, and views), and if special effects or media (videos, photos, etc.) or viral subjects such as reality stars or shaming were utilized.

## Results

Sixty-three percent of the posts on Instagram originated with plastic surgeons, compared to 18% on Facebook and only 13% on YouTube (*P*< 0.01); combined, plastic surgeons’ posts constituted 31% of the total posts, while 49% of posts were published by commercial companies (*P*<0.01). Most Instagram posts were self-promotional (83%), in comparison to Facebook (29%) or YouTube (6%) (*P*<0.01). YouTube posts were more personal in nature compared to Instagram and Facebook (39%, 7%, and 9%, respectively; *P*<0.01). Educational content accounted for only 16% of the posts (*P*<0.01).

Shaming was found in 21% of the posts, especially on Facebook (39%), and mainly related to famous public figures (25%) (*P*<0.05). Celebrity-endorsed posts received more attention in every aspect of social media currency (likes, comments, shares, and views).

The use of images of women attracts attention and was widely used in social media posts related to plastic surgery (68%). Posts that included videos (22%) were generously rewarded. Online shaming also attracted attention, mostly found in Facebook (39%), and mainly of a public figure (25%).

## Conclusions

Social media have become an important tool for self-promotion, and a perceived means to providing better customer service. This trend also applies to plastic surgeons. The study’s main insights were to use Instagram, personal stories, educational posts, videos, and other unique inputs, and to involve celebrities in the posts. In general, plastic surgeons would be wise to invest in understanding the social media communication platforms, as they have become a dominant path in the field.

# Abstract #013. Post-mild Traumatic Brain Injury: Pain Chronification Timeline and Distribution

**Published:** 2019-01

**Authors:** Pora Kuperman, Noam Bosak, Yelena Granovsky, Michal Granot, Hany Bahouth, Shiri Fadel, Hen Ben-Lulu, Avihu Marco, Chen Buxbaum, Elliot Sprecher, David Yarnitsky

**Affiliations:** 1 The Ruth & Bruce Rappaport Faculty of Medicine, Technion–Israel Institute of Technology, Haifa, Israel; 2 Department of Neurology, Rambam Health Care Campus, Haifa, Israel; 3 Faculty of Social Welfare and Health Sciences, University of Haifa, Haifa, Israel; 4 Trauma Center, Rambam Health Care Campus, Haifa, Israel

## Background

The 2004 WHO Collaborating Centre Task Force on mild traumatic brain injury (mTBI) reported that overall most patients recovered 3 months to 1 year post-injury. Additionally, the 2014 International Collaboration on mTBI Prognosis found that more than 50% of mTBI patients post-car accident still reported symptoms such as headache, neck pain, or sleep disturbances after 1 year. Current work, however, offers no further delineation of pain change and/or progression within this time period.

## Study Aims

The aim of this study was to investigate area-of-injury pain distribution in two phases: very-early acute (<72 hours) and chronic phases (<12 months), as well as the chronicity timeline of mTBI post-car accident participants.

## Materials & Methods

The study cohort consisted of 103 consecutive patients (age range 19–67 years, 43F), recruited between March 2016 and September 2017, who provided pain scores at baseline and for at least 6 months post-accident.

A reciprocal time regression model was used. Distribution plots were calculated for head and neck pain at baseline and 12 months post-accident; 12-month distributions were found to be skewed with a long tail at higher pain levels.

## Results

Visual examination of the model suggested pain stabilization occurring between months 2 and 4.

Head and neck pain distribution at baseline showed similar mean (head, 50.9±28.8; neck, 55.2± 27.7) and median (head, 50; neck, 60) values, suggesting a more normally distributed plot.

Both sites (*n*=84) had a median value of 0 at 12 months (Numerical Plain Scale [NPS] 0: head *n*=47, neck *n*=49). However, mean pain scores were clinically significant (>20, 0–100 NPS) (head, 25.6±33.8; neck, 23.9±32.5), whereas 27 individuals scored pain values of 50 to 100 (NPS) in their head and 25 in their neck, suggesting non-normally distributed plots (Shapiro–Wilk *P*<0.001).

## Conclusions

Area-of-injury pain chronification in mTBI post-accident patients lends support to the latest guidelines for WHO’s International Classification of Diseases, which holds that chronic pain is persistent or recurring pain lasting longer than 3 months. When examined in greater depth, although at 1 year 57% of the cohort was entirely pain-free, 72% of those who did have pain reported moderate–severe levels (≥50 NPS), creating what seems to be an “all or nothing” pain dichotomy. This distribution is unlike persistent post-surgical pain, wherein nearly 60% of patients are pain-free and only 40% of participants in pain reported moderate–severe pain (≥30 NPS). One potential explanation for this difference rests on the traumatic nature of the mTBI injury, which can result in concurrent “polytrauma.” This dichotomy warrants further investigation, particularly to determine factors that influence high area-of-injury pain at the 1-year juncture.

# Abstract #014. Leave Your Underwear On! A New Approach for Patients Going to Surgery

**Published:** 2019-01

**Authors:** Pazit Shachar Gelber, Adnan Halil

**Affiliations:** 1 Department of the Operating Room, and Pre-anesthesia Clinic, Rambam Health Care Campus, Haifa, Israel; 2 Post-anesthesia Care Unit, Rambam Health Care Campus, Haifa, Israel

## Background

For patients, going to the operating room without underwear often evokes feelings of discomfort, impaired privacy, and lack of dignity. The opinions regarding this issue are divided, and some see it as a “myth” rooted in a culture of work, with no justifiable rationale.

## Study Aims

This study assessed the attitudes of health-care personnel regarding whether or not patients should enter the operation room (OR) with underwear, as a basis for consideration of future hospital policy changes.

## Materials & Methods

A survey was distributed to doctors and nurses working in the OR, post-operative recovery room, and surgical departments. They were completed and anonymously submitted. Analysis relied on descriptive information and analytical statistics (*t* test, logistic regression).

## Results

Most of the 327 respondents (79%) thought there was no need for patients to remove underwear for surgeries of short duration, patients with low surgical risk, or surgeries not involving the lower genital area or upper thighs. Reasons for justifying underwear removal were: risk of surgical field flare due to diathermy, increased risk for pressure ulcers during prolonged surgery, the need to expose the body during life-threatening situations, and to prevent infections. Only 15% of those supporting patients entering the OR with underwear thought that disposable underwear should be offered to patients (*P*=0.001, odds ratio=0.33, 95% confidence interval 0.19–12).

With regard to the patients’ feelings of discomfort, nurses reported a higher degree of patient discomfort when underwear was removed before surgery, than did doctors (76% vs. 63%, respectively; *P*=0.002).

With regard to where underwear should be removed, 33% of surgeons compared to 10% of anesthesiologists (*P*=0.02) preferred that underwear be removed in the surgical departments.

With regard to responsibility for asking patients to remove their underwear, 53% of the surgical department nurses believed this was the responsibility of nurses in the OR receiving room, compared to 34% of the OR nurses (*P*=0.007).

## Conclusions

Health-care personnel are willing to make changes in the guidelines regarding the removal of patients’ underwear before entering the OR. The surgical area, patient’s health status, and anesthesiologist/surgeon requirements are the most important factors influencing these changes. A multidisciplinary and multi-departmental working group should be convened to develop clear and appropriate guidelines. Further research is needed to assess patient attitudes before changing the guidelines.

# Abstract #015. The Adequacy of Colonoscopic Bowel Preparation Is Highly Dependent on the Amount of Liquid Consumption

**Published:** 2019-01

**Authors:** Yuri Gorelik, Eisa Hag, Tomer Hananya, Ronit Leiba, Elizabeth Half

**Affiliations:** 1 Department of Internal Medicine D, Rambam Health Care Campus, Haifa, Israel; 2 Gastroenterology Institute, Rambam Health Care Campus, Haifa, Israel; 3 The Ruth & Bruce Rappaport Faculty of Medicine, Technion–Israel Institute of Technology, Haifa, Israel; 4 Department of Epidemiology, Rambam Health Care Campus, Haifa, Israel

## Background

The efficacy of colonoscopy is highly dependent on the adequacy of the pre-procedural bowel preparation. Numerous studies have identified multiple characteristics and bowel preparation variables associated with increased risk of inadequate bowel preparation (IBP).

## Study Aims

We sought to identify risk factors for IBP in an effort to recommend a simple modification that can potentially increase the rate of successful preparation.

## Materials & Methods

A prospective observational study of adult outpatients undergoing colonoscopy was performed. Subjects filled out a questionnaire with multiple patient and preparation-associated parameters. Adequacy of bowel preparation was graded according to the Boston Bowel Preparation Scale. Adequately and inadequately prepared subject groups were compared. Multivariate regression analysis was performed for variables significantly different between the groups. Additional analysis of select variables was performed.

## Results

A total of 1,172 subjects were analyzed; mean age was 55.8 years, and 52.8% were males. The IBP rate was 19.3%. Subjects with IBP were significantly older and weighed more. Compared to subjects with an academic education, subjects with less than 12 years of education had a significantly higher risk for IBP (odds ratio [OR]: 3 [95% CI 1.79–5]). Diabetes (OR: 2.26 [95% CI 1.63–3.14]), psychiatric diseases (OR: 2.1 [95% CI 1.2–3.5]), and use of proton pump inhibitors (OR: 1.95 [95% CI 1.3–2.9]), anti-hypertensives (OR: 1.78 [95% CI 1.79–5]), and statins (OR: 3 [95% CI 1.79–5]) were associated with IBP.

An interval of more than 8 hours between end of preparation to colonoscopy conferred a significantly higher risk for IBP (OR: 1.7 [95% CI 1.3–2.3]). Rate of IBP consistently decreased with an increase in fluid consumption. In comparison to patients who drank 7–10 cups of liquids, patients who drank less than 7 were at significantly higher risk for IBP (OR: 3 [95% CI 1.79–5]). Moreover, out of 66 subjects who drank more than 10 cups with each preparation packet, only one had inadequate preparation (OR: 0.1 [95% CI 0–0.4]). Multivariate analysis showed that only weight, presence of diabetes, psychiatric disease, volume of fluids consumed, and time interval to colonoscopy were significantly associated with the risk for IBP. Only 41 out of 486 (8.4%) subjects who consumed more than 8 cups with a time interval to colonoscopy of less than 8 hours failed bowel preparation.

## Conclusions

Along with previously suggested risk factors for IBP, we have shown that fluid intake in addition to the time interval to colonoscopy are easily modifiable parameters that can substantially affect the rate of IBP.

# Abstract #016. PAX8 Mechanism of Action in Endometrial Transformation

**Published:** 2019-01

**Authors:** Basem Fares, Ruth Perets

**Affiliations:** 1 Clinical Research Institute at Rambam, Rambam Health Care Campus, Haifa, Israel; 2 The Ruth & Bruce Rappaport Faculty of Medicine, Technion–Israel Institute of Technology, Haifa, Israel

## Background

Endometrial carcinoma (EC), cancer of the lining of the uterus, is the most common gynecological cancer and the fourth-most common cancer in women worldwide. EC subtypes include endometrioid adenocarcinoma (the most frequent type), uterine papillary serous carcinoma (UPSC), uterine clear-cell carcinoma, and several other rare forms. UPSC, a prototypical Type II EC, is histologically and molecularly similar to high-grade ovarian cancer. It is characterized by p53 mutations and copy number variations. PAX8 is an essential transcription factor during embryonic development of the Müllerian duct, which is the precursor of the epithelial lining of female reproductive organs, and is expressed in nearly 100% of UPSCs.

## Study Aims

The main objective of this project was to study the role of PAX8 in cellular proliferation of endometrial cancer.

## Materials & Methods

We knocked down (KD) PAX8 using RNA interference. PAX8 KD was confirmed using western blot analysis and immunofluorescent staining, and EC cell viability was measured using cell proliferation assays. The effect of PAX8 KD on cell cycle progression and apoptosis was analyzed using fluorescent activated cell sorting (FACS) and western blot analysis.

## Results

PAX8 silencing in EC cells decreased cell viability; we showed that this effect was due to apoptosis of cancer cells. We showed that, similarly to our previous finding in ovarian cancer, in UPSC PAX8 regulates a mutant GOF p53, and this effect can mediate its pro-proliferative effect.

## Conclusions

We show here for the first time that PAX8 plays an essential anti-apoptotic role in UPSC.

# Abstract #022. Homozygous Mutation in Glutamyl-tRNAGln Amidotransferase Subunit C Causes a Lethal Metabolic Phenotype with Hypertrophic Cardiomyopathy

**Published:** 2019-01

**Authors:** Alina Kurolap, Nadine Damouny Naoum, Sara Palacios-Zambrano, Adi Mory, Tamar Paperna, Rafael Garesse, Hanna Mandel, Yaniv Zohar, Miguel A. Fernández-Moreno, Hagit Baris Feldman

**Affiliations:** 1 The Genetics Institute, Rambam Health Care Campus, Haifa, Israel; 2 The Ruth & Bruce Rappaport Faculty of Medicine, Technion–Israel Institute of Technology, Haifa, Israel; 3 Departamento de Bioquímica, Instituto de Investigaciones Biomédicas “Alberto Sols” UAM-CSIC and Centro de Investigación Biomédica en Red en Enfermedades Raras (CIBERER), Madrid, Spain; 4 Facultad de Medicina, Universidad Autónoma de Madrid, Madrid, Spain; 5 Instituto de Investigación Sanitaria Hospital 12 de Octubre (imas12), Madrid, Spain; 6 Metabolic Unit, Rambam Health Care Campus, Haifa, Israel; 7 Institute of Pathology, Rambam Health Care Campus, Haifa, Israel

## Background

Precise mRNA-to-protein translation is essential for organism function. In the mitochondria, each tRNA is paired with its cognate amino acid by 19 aminoacyl-mt-tRNA synthetase enzymes. Gln-tRNA^Gln^ assembly is unique, requiring an alternate two-step pathway. In this process, mt-tRNA^Gln^ is first misacylated with glutamate by Glu-tRNA synthetase (EARS2), which is subsequently transamidated to glutamine, forming Gln-tRNA^Gln^. The last step is executed by the glutamyl-tRNA amidotransferase complex of three subunits: GatCAB.

## Study Aims

The aim of this study was to uncover the genetic defect leading to a hypertrophic cardiomyopathy syndrome with severe lactic acidosis and early lethality in affected infants from two reportedly unrelated families of Druze origin.

## Materials & Methods

Trio whole-exome sequencing (WES) was performed for the first family. Sanger sequencing was used for diagnosis in the second family and for segregation analysis. Restriction analysis was used to establish variant prevalence in the Druze village of the proband in the first family. Immunoblotting and protein synthesis studies were performed on patient fibroblasts.

## Results

Whole-exome sequencing analysis revealed a homozygous missense variant in *GATC* (c.233T>G; p.Met78Arg) that encodes subunit C of the glutamyl-tRNA amidotransferase (GatCAB) complex. This variant was not observed in general population databases (i.e. gnomAD) or the Genetics Institute internal database of over 1,000 Israeli exomes. Sanger sequencing revealed the same homozygous mutation in the affected infant in the second family, and confirmed the variant’s co-segregation with the disease in both families. Population screening revealed a prevalence of 1:50 mutation carriers in the proband’s village of residence.

Western blot performed on mitochondrial protein extracts from patient cells revealed ~80% depletion in all three GatCAB complex proteins, suggesting that *GATC* p.Met78Arg affects complex stability. A strong and generalized mtDNA-encoded protein synthesis defect was observed in patient cells compared to controls, in the presence of a cytoplasmic protein synthesis inhibitor (emetine). In addition, the ability of mutant fibroblasts to recover *de novo* synthesis following mitochondrial protein translation block was deficient relative to control cells, and especially upon depletion of mt-tRNA^Gln^ charged with glutamine, suggesting that the biochemical phenotype becomes more prominent under conditions that place mt-tRNA^Gln^ charging under stress. Joint collaboration with other international groups provided evidence of human mitochondrial disease caused by bi-allelic mutations in each of the GatCAB complex subunits.

## Conclusions

Mutations affecting mitochondrial protein synthesis machinery cause a broad spectrum of fatal disorders. A decade of research into the human diseases associated with all mitochondrial aminoacylation enzymes is now complete, with this final discovery deciphering the role of GatCAB.

# Abstract #023. A Novel Missense Mutation in *ERBB4*, Encoding the erb-b2 Receptor Tyrosine Kinase 4, Underlies Early-onset Non-syndromic Hereditary End-stage Renal Disease

**Published:** 2019-01

**Authors:** Ayala Ofir, Shirley Pollack, Israel Eisenstein, Shay Tzur, Daniella Magen

**Affiliations:** 1 Molecular Medicine Laboratory, Rambam Health Care Campus, Haifa, Israel; 2 The Ruth & Bruce Rappaport Faculty of Medicine, Technion–Israel Institute of Technology, Haifa, Israel; 3 Pediatric Nephrology Institute, Ruth Rappaport Children’s Hospital, Rambam Health Care Campus, Haifa, Israel; 4 The Genomic Research Department, Emedgene Technologies Ltd, Tel Aviv, Israel

## Background

Genetic factors play an important role in the development of end-stage renal disease (ESRD), especially during childhood. More than 200 genes were discovered so far that are associated with early-onset renal disease, and more will probably be identified. *ERBB4* encodes an epidermal growth factor (EGF) receptor tyrosine kinase. The gene product is activated upon binding of neuregulins (NRGs), among others, to its extracellular ligand-binding domain, thereby inducing cellular mitogenesis and differentiation. Recent studies indicate a role for ERBB4 in kidney development, polycystic kidney disease progression, and diabetic renal nephropathy.

## Study Aims

The aim of this study was to investigate the clinical features and the molecular basis of familial renal failure of early onset in a consanguineous Palestinian family.

## Materials & Methods

Three patients with autosomal recessive ESRD and 19 of their close relatives were clinically and genetically studied. Mutation analysis was performed by whole-exome sequencing followed by conventional Sanger sequencing. For expression analysis, the identified mutation was introduced into plasmids encoding two of the predominant renal splice isoforms of *ERBB4*, Jma-Cyt1 and Jma-Cyt2. RNA expression and protein stability were measured using a cell-culture system. Cellular protein localization was studied by immunofluorescence.

## Results

Phenotypic evaluation indicated isolated renal failure of early onset and unknown cause, propagating to ESRD during adolescence. Mutation analysis revealed a novel homozygous missense mutation (c.3813G>T;p.E1287D) in *ERBB4* in all three affected siblings. The mutation segregated completely with the disease phenotype in all studied participants. RNA expression analysis using real-time polymerase chain reaction (PCR) revealed similar wild-type and mutant RNA levels. Nevertheless, the E1287D-mutant protein level was dramatically reduced as compared to that of the wild-type isoforms.

Protein stability analysis revealed a significantly diminished half-life of the E1287D-mutant protein of less than 2 hours, as compared to ~7 hours in the wild-type isoforms, suggesting instability and massive degradation of the E1287D-mutant protein. Immunofluorescence analysis showed a remarkable decrease in E1287D-mutant protein expression, accompanied by aberrant intracellular accumulation within the endoplasmic reticulum, as opposed to normal plasma membrane targeting of the wild-type isoforms.

## Conclusions

We identified a novel homozygous mutation in *ERBB4* associated with familial, non-syndromic ESRD with autosomal recessive inheritance. Expression and localization analyses indicate instability and cellular mistargeting of the mutant protein. Our findings suggest a major role for *ERBB4* in the pathogenesis of familial ESRD, thereby enabling genetic counseling and prenatal diagnosis in affected families. The molecular mechanism whereby *ERBB4* disruption underlies ESRD remains to be identified.

# Abstract #025. Point of Care Ultrasound (POCUS) Learning Center

**Published:** 2019-01

**Authors:** Nira Beck-Razi, Diana Gaitini, Ziva Besser, Dorit Halevi, Marcia Javitt

**Affiliations:** 1 Department of Medical Imaging, Rambam Health Care Campus, Haifa, Israel; 2 The Ruth & Bruce Rappaport Faculty of Medicine, Technion–Israel Institute of Technology, Haifa, Israel; 3 Rambam Knowledge Center, Rambam Health Care Campus, Haifa, Israel

## Background

Core knowledge and practical training are needed for performance and interpretation of point of care ultrasound (POCUS), an essential tool for urgent and emergent clinical decision-making at the bedside.

## Study Aims

This study was aimed at building a state-of-the-art POCUS blended learning center.

## Materials & Methods

From 2013 to 2018, Rambam provided POCUS training courses for medical students, practicing physicians, and first responders, using blended learning that combined: (1) frontal lectures on core knowledge; (2) hands-on skills training on phantoms, live models, and patients; (3) trainee evaluations using either the web-based interactive website (kahoot.com), or written tests.

Currently under development are: (1) a dedicated computer-based interactive learning software program; (2) 3D virtual reality simulations based on real cases; (3) extended metrics for trainee performance; and (4) competency certification with skills-based testing.

## Results

Attendee course evaluations from two previous courses (May 2017 and February 2018) were analyzed. Thirty-five doctors attended the 2017 course, of whom 29 were from internal medicine, 4 from the emergency room, 1 from oncology, and 1 from the Israeli Defense Forces. Thirty physicians and first responders attended the 2018 course—20 residents and 10 specialists as follows: 12 from internal medicine, 7 from surgery, 5 from an emergency medical team, 5 from the emergency room, and 1 from the intensive care unit. A Likert scale ranked from lowest (1) to highest (5) was used to evaluate satisfaction with the course. For the first course, a mean score of 4.76±0.36 was given for the hands-on session, with a score of 4.73±0.39 for the second course. These scores demonstrate high levels of attendee confidence and satisfaction.

## Conclusions

This state-of-the-art blended learning center promotes informed and skilled use of POCUS for improved user confidence. Further pre- and post-test evaluations are being designed to evaluate the impact of this course on the learners’ fund of knowledge and skills in support of better quality of care and patient outcomes.

# Abstract #029. BDNF Protein Levels and Gene Polymorphism: A Potential Biomarker for Chemotherapy-induced Peripheral Neuropathy in Lymphoma and Myeloma Patients

**Published:** 2019-01

**Authors:** Netanel Horowitz, Sami Giryes, Roni Nasser, Rivka Sharon, David Azoulay

**Affiliations:** 1 Hematology and Bone Marrow Transplantation Institute, Rambam Health Care Campus, Haifa, Israel; 2 Department of Internal Medicine B, Rambam Health Care Campus, Haifa, Israel; 3 Hematology Laboratory and Blood Bank, Galilee Medical Center, Naharia, Israel; 4 The Azrieli Faculty of Medicine, Bar-Ilan University, Safed, Israel

## Background

Chemotherapy-induced peripheral neuropathy (CIPN) is a major and dose-limiting side effect in hematological malignancies (HM). Brain-derived neurotrophic factor (BDNF) is a neuronal growth factor essential for nervous system maintenance. The role of BDNF protein levels or gene polymorphism in CIPN development is unknown.

## Study Aims

We evaluated the role of BDNF gene single nucleotide polymorphism (Val66Met-SNP) and peripheral blood (PB) BDNF in CIPN-developing non-Hodgkin lymphoma (NHL) and multiple myeloma (MM) patients.

## Materials & Methods

Forty-five patients (33 NHL and 12 MM) were included in this study. CIPN was assessed at diagnosis, and during vincristine- or bortezomib-based therapy, using the Total Neuropathy Score (TNSr) and Functional Assessment of Cancer Therapy/Gynaecologic Oncology Group-Neurotoxicity (FACT-GOG-NTx). Depression was assessed using PHQ9 questionnaire. Val66Met-SNP and BDNF protein levels were quantified with Sanger sequencing and ELISA, respectively.

## Results

A significant inverse correlation was revealed between pre-treatment BDNF protein levels and maximal TNSr, FACT-GOG-NTx, and PHQ9 scores in both genotypes (*r*=0.608, *P*=0.0001; *r*= 0.607, *P*=0.0001; and *r*=0.461, *P*=0.004, respectively). Such correlations were not observed with the number of platelets, which are known to be the main BDNF compartment in PB. Met-BDNF patients demonstrated significantly lowered maximal FACT-GOG-NTx and PHQ9 scores compared to Val-BDNF patients (4.77±2.58 vs. 8.03±4.55, *P*=0.02; and 3.31±2.32 vs. 7.93±5.32, *P*=0.004, respectively). These data suggest that patients with high vulnerability to CIPN might be identified by pre-treatment lower protein levels of BDNF. In addition, the Met-BDNF polymorphism was associated with attenuated perception of CIPN symptoms.

## Conclusions

Determination of BDNF protein levels and gene polymorphism before chemotherapy initiation might be a useful new tool for CIPN risk assessment and pre-treatment dose modification. These data should be validated in larger studies and for other anti-cancer agents.

# Abstract #031. Endothelial Progenitors Prevent Bisphosphonates-induced Osteonecrosis by Stimulating Angiogenesis and Improving Wound Healing

**Published:** 2019-01

**Authors:** Ofri Doppelt, Gal Cohen, Reut Book, Rina Elimelech, Tal Tamari, Hadar Zigdon

**Affiliations:** The Clinical Research Institute at Rambam, Rambam Health Care Campus, Haifa, Israel

## Background

Medication-related osteonecrosis of the jaw (MRONJ) is a serious adverse effect of anti-resorptive and anti-angiogenic drugs, presenting a chronic wound in the oral mucosa that exposes necrotic jaw bone, usually following tooth extraction. In a previous MRONJ rat model, we showed a significant decrease in soft tissue vascularization that was associated with exposed necrotic bone in 50% of rats treated with zoledronic acid (ZOL, a potent antiresorptive drug) and dexamethasone (DEX). We hypothesized that impaired soft tissue wound healing and reduced vascularization play a role in MRONJ pathogenesis, and that increasing soft tissue vascularization before tooth extraction may prevent MRONJ.

## Study Aims

Our aim was to assess the potential of pro-angiogenic endothelial progenitor cells (EPCs) to prevent MRONJ in an *in vitro* and *in vivo* study.

## Materials & Methods

Sixteen Lewis rats were treated with a subcutaneous injection (s.c.) of ZOL (7.5 μg/kg) and DEX (1 mg/kg) once a week, for 11 weeks. After 1 week, stem cells were injected to the gingiva around the first maxillary molars. Rats were randomly divided to three treatment groups: EPCs, mesenchymal stem cells (MSCs), and medium (control). At 3 weeks, bilateral first maxillary molars were extracted, and healing was allowed for 8 weeks before sacrifice. Healing of the extraction site was evaluated clinically and histologically in order to detect necrotic bone. Serum VEGF and blood vessel density were measured. In addition, human gingival fibroblasts were cultured with EPC-conditioned media (EPC-CM)/endothelial growth media/DMEM for 72 h, followed by incubation with ZOL+DEX 10 μM for an additional 72 h. The proliferation and migration abilities were assessed by 2,3-Bis-(2-methoxy-4-nitro5-sulfophenyl)-2H-tetrazolium-5-carboxanilide salt (XTT) proliferation and scratch wound healing assays.

## Results

Eighty percent of the extraction sockets in the EPCs and medium groups showed normal wound healing. However, 50% of the extraction sites in the MSC group showed open wound and exposed bone. The EPCs had elevated serum VEGF levels (*P*=0.05) and increased blood vessel density (*P*=0.01). Additionally, fibroblasts treated with EPC-CM following exposure to ZOL and DEX demonstrated improvement proliferation and scratch wound healing, compared to standard medium (*P*=0.007, *P*=0.023).

## Conclusions

We concluded that a local injection of EPC before tooth extraction increased soft tissue vascularization and prevented MRONJ. Moreover, EPCs secretome improved wound healing by reducing the cytotoxic effects of ZOL and DEX on fibroblasts.

# Abstract #033. How Many People Search for Their Symptoms Online?

**Published:** 2019-01

**Authors:** Irit Hochberg, Raviv Alon, Elad Yom-Tov

**Affiliations:** 1 Endocrinology, Diabetes, and Metabolism Institute, Rambam Health Care Campus, Haifa, Israel; 2 The Ruth & Bruce Rappaport Faculty of Medicine, Technion–Israel Institute of Technology, Haifa, Israel; 3 Microsoft R&D Center, Herzliya, Israel

## Background

Data collected in surveys in the USA indicate that most internet users search online for information on medical symptoms they experience, and approximately one-third self-diagnose using information they find on the internet. Yet, surveys have limitations and biases, and the true rates of search engine querying for medical symptoms before diagnosis are unknown.

## Study Aims

This study set out to estimate the frequency in which internet users query their symptoms prior to diagnosis.

## Materials & Methods

We studied the Bing search queries made in the USA over a 1 year period, with goal of finding users who explicitly stated that they had been diagnosed with one of 20 medical conditions which are usually diagnosed following symptom appearance, and not screened for nor diagnosed as part of routine medical tests. We then assessed how many of these people searched online, prior to diagnosis, for the symptoms related to their medical condition. To test the hypothesis that people are more likely to search online if symptoms are unfamiliar, we additionally administered a survey to a small group of people, to quantify whether laypeople recognized symptoms associated with these conditions.

## Results

On average, 15.5%±8.4% of users searched online for their symptoms. A longer period of time between the first query regarding a symptom and the diagnosis was associated with an increased likelihood of queries on symptoms (ρ=0.6, *P*=0.005). Symptom-condition associations with which laypersons were unfamiliar were also a predictor for symptom searches (ρ=−0.47, *P*=0.08). There was a 14% higher likelihood for an online search if the symptom was worrying.

## Conclusions

Our results suggest that only a minority of patients diagnosed with symptomatic medical conditions search the internet for their symptoms prior to diagnosis. This finding has important implications for systems that screen for medical conditions based on people’s internet searches.

# Abstract #034. Cervical Insufficiency Postpartum: The Missing Chapter

**Published:** 2019-01

**Authors:** Roy Lauterbach, Emad Matanes, Chen Ben David, Omer Mor, Revital Linder, Adi Cohen-Halbertal, Yaniv Zipori, Zeev Weiner, Ron Beloosesky, Yuval Ginsberg

**Affiliations:** Division of Obstetrics and Gynecology, Rambam Health Care Campus, Haifa, Israel

## Background

Little is known regarding cervical recovery postpartum. To date, very few studies have addressed the issue of cervical recuperation, and common practice dictates that the uterine cervix returns to its pre-pregnancy state up to 6 weeks postpartum. This notion is mere speculation and is not evidence-based.

## Study Aims

The main objective of this study was to evaluate differences in cervical recovery following preterm compared to term vaginal delivery. Secondary objectives included differences in cervical recovery following various obstetric circumstances.

## Materials & Methods

This was a prospective cohort study of partituents postpartum undergoing transvaginal cervical length measurement (GE730 expert) by a single observer at three different time periods (8, 24, 48 hours postpartum) between July 2016 and August 2018. All women after vaginal delivery or cesarean section (CS) at full dilation were eligible for recruitment. Results were adjusted for demographic characteristics, obstetric history, parity, delivery outcomes, and induction mode.

## Results

The cohort included 714 women. The average cervical length 48 hours postpartum, following term deliveries, was 35.2±3.5 mm.

### Parity

The most rapid cervical recovery was demonstrated following the second delivery (39.7± 2.5 mm). A significant delay in cervical recovery was observed from the fourth delivery and above (27.8± 2.7 mm; *P*=0.013).

### Preterm delivery

Women delivered before 34 weeks’ gestation (*n*=119) demonstrated abnormal cervical recovery when compared to deliveries between 34–37 (*n*=97) weeks and term deliveries (*n*=498; 17.2±1.9 mm vs. 32.4±3.6 mm vs. 35.2± 3.5 mm, respectively; *P*<0.0001).

### Mode of induction

In total 156 patients underwent medical induction (dinoprostone or oxytocin), and 88 underwent mechanical induction by cervical ripening balloon (CRB). Abnormal cervical recovery was demonstrated in the CRB group (26.3±1.9 mm vs. 35.2±2.7 mm, respectively; *P*= 0.012).

### Cesarean section

Thirty-eight patients underwent emergent CS at full dilatation. Significant pathological cervical recuperation was demonstrated following emergent CS due to arrest of descent compared to emergent CS due to non-reassuring fetal heart rate and spontaneous vaginal deliveries (27.4± 2.5 mm vs. 30.4±1.6 mm vs. 35.2±3.5 mm respectively; *P*=0.018).

## Conclusions

This study provides a novel insight regarding cervical recuperation postpartum. For the first time, nomograms of postpartum cervical recovery were established. Early spontaneous preterm delivery is associated with pathological cervical recovery, which might imply “true” cervical insufficiency. Increased parity, mechanical inductions, and emergent CS due to prolonged second stage may cause damage to the cervix. Although the clinical implications of delayed postpartum cervical recuperation have not been elucidated, it might imply future cervical insufficiency and possible increased risk of preterm birth.

# Abstract #035. Optimizing Urine Composition for the Treatment of Urothelial Cancer with a Controlled Release System Containing Cisplatin

**Published:** 2019-01

**Authors:** Lena Ilan, Ahmad Kabha, Eyal Zussman, Gilad Amiel

**Affiliations:** 1 Department of Urology, Rambam Health Care Campus, Haifa, Israel; 2 The Clinical Research Institute at Rambam, Rambam Health Care Campus, Haifa, Israel; 3 Faculty of Mechanical Engineering, Technion–Israel Institute of Technology, Haifa, Israel

## Background

Urothelial cancer is the fourth most common cancer in men and eighth most common in women. It is considered the most expensive cancer to treat due to the high recurrence rate (>50%). In most (85%) patients it appears in the bladder, and in others in the upper urinary tract including the renal pelvis and ureter. The mainstay treatment for advanced disease is a combination of cisplatin-based chemotherapy in addition to surgery or external beam radiation. Cisplatin is given intravenously with many side effects and complications, including damage to renal function, anemia, lymphopenia, and nausea, that limit many patients’ ability to complete the treatment protocol. These side effects can be significantly reduced or eliminated by local application of the chemotherapeutic agent.

## Study Aims

This study was aimed at analysis of interaction between cisplatin and urine, toward development of a controlled release system containing cisplatin in the genito-urinary system for the treatment of urothelial cancer.

## Materials & Methods

We treated bladder cancer cell lines in standardized artificial urine solution (AUS) with cisplatin. We used sulforhodamine B colorimetric assay to measure the efficacy and toxicity of cisplatin at different AUS concentrations and at different time points.

## Results

As expected, both AUS and cisplatin are toxic for T-24 and HTB-9 bladder cancer cell lines. When combining AUS and cisplatin, we found that AUS reduced cisplatin activity in a concentration-dependent manner. In the presence of 10% AUS, the number of cells surviving 72 hours of treatment was 3.7 times higher than in samples treated with cisplatin alone. The addition of 40% AUS increased cell survival by more than eight times compared to the non-AUS sample. While analyzing the factors causing cisplatin inhibition by AUS, we demonstrated that among the chemical components of AUS, isolated NaCl and creatinine have a small inhibitory effect, whereas their combination increased cell survival threefold compared to control. An additional small but significant effect was obtained by changing AUS pH from acidic to neutral.

## Conclusions

Most of the cisplatin administered intravenously is excreted through the urine within 12 hours of administration. We developed a cisplatin-based controlled release system for the genito-urinary tract for treatment of urothelial cancer. Since the composition of human urine can be changed with IV and oral medications, understanding the interaction of cisplatin with urine components and pH will allow us to adjust the degradation rate of the cisplatin-containing agent and optimize patient urine composition to maximize treatment efficiency.

# Abstract #037. The Association between Endothelial Protein C Receptor (EPCR) A6936G Polymorphism and EPCR Expression in Tumor Cells from Lung Adenocarcinoma Patients

**Published:** 2019-01

**Authors:** Therese Shehadeh Mabariki, Edmond Sabo, Ran Kremer, Ori Haverfeld, Galit Sarig

**Affiliations:** 1 Immunology Laboratory, Rambam Health Care Campus, Haifa, Israel; 2 Hematology Laboratory, Rambam Health Care Campus, Haifa, Israel; 3 The Ruth & Bruce Rappaport Faculty of Medicine, Technion–Israel Institute of Technology, Haifa, Israel; 4 Department of Thoracic Surgery, Rambam Health Care Campus, Haifa, Israel; 5 Department of Thoracic Surgery, Rambam Health Care Campus, Haifa, Israel

## Background

Thrombotic complications and hypercoagulation in cancer are associated with tumor progression and poor patient outcome. The protein C pathway and the endothelial protein C receptor (EPCR) play major roles in anticoagulant and cytoprotective mechanisms in malignancy. The EPCR A6936G polymorphism is associated with high soluble EPCR (sEPCR) plasmatic levels, which is a moderate risk factor for thrombotic events and has recently been found to be associated with prolonged survival time in advanced non-small cell lung carcinoma (NSCLC) patients. The mechanism of sEPCR and EPCR A6936G polymorphism association with EPCR expression on tumor cells and tumor progression has not been clarified yet.

## Study Aims

This study aimed to determine the association between EPCR A6936G polymorphism and EPCR expression levels on tumor cells from early-stage lung adenocarcinoma patients. It also examined the association between EPCR A6936G polymorphism and EPCR expression levels with stage of disease, time to disease progression, and survival time in these patients.

## Materials & Methods

DNA samples were extracted from 293 archival formalin-fixed paraffin-embedded (FFPE) tissue blocks of lung adenocarcinoma patients. Fluorescent resonance energy transfer (FRET) real-time polymerase chain reaction (PCR) and Sanger sequencing reactions were used to determine EPCR A6936G polymorphism. Sections from 83 lung adenocarcinoma patients were strictly matched according to age, gender, and stage of disease between sections from patients with EPCR 6936AG/GG polymorphism vs. patients with EPCR 6936AA polymorphism, then immunohistochemically stained with anti-EPCR antibodies. Quantitation of EPCR expression and co-localization was evaluated by pathologist scoring, which incorporated both intensity and extent scores.

## Results

The EPCR expression level in tissue sections with EPCR 6936AA polymorphism were significantly higher compared to tissue sections with AG/GG polymorphism (mean membrane score 1.0 vs. 0.6, *P*=0.038; median cytoplasm extent 60% vs. 30% staining, *P*=0.048). Patients whose tissue sections were strongly immunostained with the EPCR antibody had shorter survival time than those who had less EPCR expression on their tumor cells (35±9 vs. 51±5 months, *P*=0.044). Most patients with EPCR 6936AG/GG (72%) were diagnosed at the lower stage of lung adenocarcinoma, with a threefold probability of being at the lower stage (<2a) than at higher stages of the disease (*P*=0.03).

## Conclusions

EPCR 6936AG/GG polymorphisms were found to be associated with low expression levels of EPCR on lung adenocarcinoma cells and with low stages (1a, 1b) of disease. Low expression levels of EPCR were associated with prolonged survival time in the studied lung adenocarcinoma patients. These results and the role of EPCR in malignancy should be further studied.

# Abstract #039. The Role of Thorough Clinical Evaluation in an Inherited Arrhythmia Clinic in the Prevention, Diagnosis, and Prognosis of Non-ischemic Sudden Cardiac Death

**Published:** 2019-01

**Authors:** Miry Blich, Hodaya Oron, Wisam Darawsha, Mahmoud Suleiman, Lior Gepstein, Monther Boulos

**Affiliations:** 1 Department of Cardiology, Rambam Health Care Campus, Haifa, Israel; 2 The Ruth & Bruce Rappaport Faculty of Medicine, Technion–Israel Institute of Technology, Haifa, Israel

## Background

Determining the pathogenesis of sudden cardiac death (SCD) not of ischemic heart disease is crucial for management, prognosis, and SCD prevention in other family members. Specialized inherited arrhythmia clinics were established to optimize diagnosis, management, and prevention of SCD.

## Study Aims

Our aim was to analyze the effectiveness of thorough clinical evaluation in a specialized unit.

## Materials & Methods

The study included 104 families with at least one index case of SCD, age 29±16 years. Survivors were studied with electrocardiography (ECG), 12 lead Holter with and without high leads, exercise testing, cardiac imaging, familial study, and genetic testing. Deceased probands were studied using the clinical evaluation before death, postmortem findings, familial evaluation, and genetic testing when available.

## Results

The most prevalent diagnoses were inherited channelopathies in 46/104 (44%) cases. Patients (*n*=23; 22%) were diagnosed with cardiomyopathy. The cause of SCD was not associated with a genetic disease only in 12 patients. After a thorough clinical evaluation in a specialized clinic, a positive diagnosis was increased from 32% to 80% (*P*<0.001).

Using the Wilcoxon–Mann–Whitney test we found that the most useful modalities to reach the diagnosis were ECG (effect size 0.5, *P*<0.001), 12-lead Holter (effect size 0.33, *P*=0.001), and family screening (effect size 0.4, *P*=0.001). Genetic testing was performed in 69 index cases with a positive result in 37 (54%). Of 421 (65%) family members who underwent clinical evaluation in the clinic, 153 (36%) had a positive result, 130 family members (31%) carried a pathogenic mutation found in the proband, and 127 (83%) family members were followed in the clinic and medically treated. Twenty-five (15%) family members underwent implantable cardioverter-defibrillator (ICD) implantation, and 9 (40%) experienced appropriate ICD shock due to life-threatening ventricular arrhythmia. In a follow-up of 6.3±5 years, one family member who refused ICD had SCD; another family member died with ICD, most probably not due to cardiac cause. This is in contrast to family members who were not followed in the clinic, of whom 13 (6%) experienced SCD (*P*<0.001). During follow-up, 29 probands experienced 58 ventricular arrhythmic events that were successfully treated by appropriate ICD shock.

## Conclusions

There is a strong likelihood of reaching a diagnosis in SCD after a rigorous protocol in a specialized unit. The most powerful tools were 12-lead Holter ECG and family screening. Family screening has a major role in the diagnosis and prevention of SCD.

# Abstract #042. An Efficient Single-session Spatial Skill Trainer for Robot-assisted Surgery

**Published:** 2019-01

**Authors:** Roy Lauterbach, Emad Matanes, Liel Luko, Avi Parush, Lior Lowenstein

**Affiliations:** 1 Department of Obstetrics and Gynecology, Rambam Health Care Campus, Haifa, Israel; 2 The Faculty of Industrial Engineering, Technion–Israel Institute of Technology, Haifa, Israel

## Background

Robotic surgery has been implemented in the field of gynecology in the past 10 years. Training young surgeons is challenging due to the high cost of equipment. The da Vinci operating system offers simulator training options for young surgeons, and spatial training sessions have proven in the past to have high efficacy.

## Study Aims

This study aimed to introduce and examine a single session of spatial skill training as an efficient means of improving surgical suturing performance in robot-assisted surgery.

## Materials & Methods

From June 2017 through January 2018, a convenience sample of 41 residents with non-robotic surgical suturing skills were randomly assigned to training and control groups, after completing two da Vinci skills simulator (dVSS) tasks to determine their baseline performance. The training group underwent computer-based simulator training of spatial skills, while the control group engaged in a neutral activity. The experiment was performed in a single tertiary university medical center in Israel.

In order to evaluate post-training performance, two dVSS tasks were performed within 2 days of training completion.

## Results

One participant failed to show up for the training and evaluation phases. Data of 40 participants were analyzed: control group *n*=20, mean age 32 years (SD=3.17); training group *n*=20, mean age 34 years (SD=1.92). Piercing after training (but not after the neutral activity) was significantly lower than before training (3.25 [SD=1.996] vs. 6.75 [SD=3.68[, respectively; *P*<0.001), reflecting an improvement of 52%. Following the experiment, the extent of tissue tearing was rated lower in the training group, *P*=0.01; it was unchanged in the control group, *P*=0.14.

## Conclusions

We demonstrated the efficiency and effectiveness of a training approach that focuses on spatial skills critical in robot-assisted surgery. We demonstrated that surgeons receiving a one-session spatial skill training immediately improved their performance of robotic surgical suturing, compared with surgeons not receiving such training.

# Abstract #043. Geographic Variation of Hysterectomy Rates in the Israeli Health Care System

**Published:** 2019-01

**Authors:** Roy Lauterbach, Ziona Haklai, Joseph Mendelovic, Gil Lavie, Lior Lowenstein

**Affiliations:** 1 Division of Obstetrics and Gynecology, Rambam Health Care Campus, Haifa, Israel; 2 Ministry of Health, Jerusalem, Israel; 3 Shaare Zedek Medical Center, Jerusalem, Israel; 4 Layd Davis Carmel Medical Center, Haifa, Israel

## Background

In 2014, the OECD published a report regarding inter-regional variation of hysterectomies in 13 countries including Israel. Variance in hospital admission rates were also reported. Israel’s Ministry of Health has set as one of its main goals reduction in differences in health care, particularly between the country’s periphery and central regions. Differences may reflect variations in patient characteristics, resource allocation, medical staff employment, expertise, and training. Advances in technology in recent decades, including laparoscopic and robotic surgeries, and the variance in their implementation demonstrate great regional variance.

## Study Aims

The aim of this study was to examine hysterectomy trends in the past decade with an emphasis on regional differences.

## Materials & Methods

This study presents data obtained from the National Hospital Discharges Database (NHDD) records maintained by the Israeli Ministry of Health. This database is continuously updated based on quarterly electronic reports from all acute care hospitals in Israel, including individual admissions, demographic characteristics, surgical approach, and length of hospitalization.

Hysterectomy rates are presented for each of the seven districts in Israel, as defined by the Ministry of Interior.

Hysterectomy rates were calculated and generally presented as rates per 100,000 women aged 25 years and over. We examined hysterectomy rates according to patient age, surgical indication (benign or malignant), and surgical approach (abdominal, vaginal, or laparoscopic). Chi-square of association was used for comparison between categorical parameters. Significance was determined for *P* value <0.05.

## Results

The study portrays the trend in hysterectomy rates as a factor of indication, surgical approach, and length of hospitalization. Significant inter-regional differences were found between seven regions of Israel, though overall there was a clear trend toward a national decrease in hysterectomy rates. A 2- to 4-fold increase in laparoscopic hysterectomies was observed, as well as a country-wide trend toward shortening hospital stay.

## Conclusions

The decline in hysterectomy rates in the past decade may be due to earlier diagnosis or new technologies, which enable additional options for expectant management. Inter-regional variations may reflect both demographic and socio-economic differences; religious factors may also affect patient medical decisions. In addition, uneven allocation of technological and human resources may have contributed to the findings.

# Abstract #044. Single versus Multi-port Robotic Assisted Sacrocolpopexy: Results from a Tertiary Single Center

**Published:** 2019-01

**Authors:** Roy Lauterbach, Susana Mustafa-Mikhail, Emad Matanes, Amnon Amit, Zeev Weiner, Lior Lowenstein

**Affiliations:** Division of Obstetrics and Gynecology, Rambam Health Care Campus, Haifa, Israel

## Background

In the past, the procedure of choice for pelvic organ prolapse (POP) repair was abdominal sacrocolpopexy (ASC). In recent years, since the introduction of laparoscopic surgery, the “gold standard” procedure became laparoscopic sacrocolpopexy (LSC). Over the past 10 years, robotic surgery has been introduced to the field of gynecology. Since then, its implementation has allowed surgeons to achieve a higher level of safety, finesse, and more aesthetic results. Single-port robotic surgery has become available for robotic assisted sacrocolpopexy (RSC) in the past 2 years.

## Study Aims

The main objective of this study was to compare the feasibility, learning curves, and short-term surgical outcomes and complications of single-port robotic assisted laparoscopic sacrocolpopexy (SP-RSC) vs. multi-port access (MP-RSC) in vaginal apex prolapse repair.

## Materials & Methods

This was a retrospective study aimed at comparing the first 52 MP-RSC and first 52 SP-RSC procedures performed at a single tertiary medical center. Primary outcomes included operative time, intra-operative bleeding volume, and length of hospitalization. Secondary outcomes included surgical complications.

## Results

A statistically significant difference in mean operative times was observed between the MP-RSC and SP-RSC groups: 206.5±39.4 min and 187.8±46.2 min, *P*=0.028, respectively. The estimated mean intraoperative bleeding volume was 35 (20–87.5) mL and 20 (10–47.5) mL, *P*=0.008, respectively. Mean operative times decreased between the first 15 and the next sequential 15 cases: in the MP-RSC group from 224.2±43.2 to 198.4±36.3 min, *P*=0.088; in the SP-RSC group from 222.4±53.1 to 161.3±28.2 min, *P*<0.001. The sequential 22 cases exhibited mixed trends. In both procedures, the decrease in console time seemed to be the main contributor to decreased operative time (from 160.3± 36.9 to 125.5±23.9 min, *P*=0.006, in the MP-RSC group; from 173.9±60 to 115.2±19.3 min, *P*=0.001, in the SP-RSC group). No statistically significant differences were observed in terms of length of hospitalization (days) and pain level at 24 hours postoperative, according to a 1–10-point visual analogue scale. Reports of adverse events were rare in both groups.

## Conclusions

MP-RSC and SP-RSC are both feasible surgical procedures with comparable learning curves. Long-term follow-up is required in order to evaluate further differences between the surgical approaches.

# Abstract #045. Lessons Learned in Rare Disease Diagnostics: Single-center Experience

**Published:** 2019-01

**Authors:** Karin Weiss, Alina Kurolap, Tamar Paperna, Adi Mory, Maya Steinberg, Tova Hershkovitz, Nina Ekhilevitch, Hagit N. Baris

**Affiliations:** 1 The Genetics Institute, Rambam Health Care Center, Haifa, Israel; 2 The Ruth and Bruce Rappaport Faculty of Medicine, Technion–Israel Institute of Technology, Haifa, Israel

## Background

The field of medical genetics has been revolutionized by the increased availability of next-generation sequencing technologies. Rare disease diagnostics have shifted from hypothesis-driven investigations to hypothesis-free approaches thanks to improved sequencing and analytic capabilities. The result has been significant improvement in the rare disease diagnosis rate. In addition, the identification of a wider phenotypic spectrum in known diseases, the discovery of novel phenotypes associated with known genes, the discovery of new rare diseases associated with novel genes not previously investigated, and of blended phenotypes due to more than one diagnosis have also been achieved. The identification of the genetic etiology in rare diseases offers a number of advantages: specific diagnosis can affect case management, treatment recommendations, and follow-up; it brings to an end the time-consuming and costly diagnostic odyssey; it facilitates family planning and counseling; and it triggers research on the disorder in question.

## Study Aims

As stated above, technological advances have revolutionized medical genetics, facilitating discovery of causative genes in numerous Mendelian disorders. Nevertheless, many cases remain undiagnosed. We report the experience of the Genetics Institute at Rambam Health Care Campus in rare disease diagnostics using whole-exome sequencing (WES).

## Materials & Methods

Phenotypic characterization of patients was performed in close collaboration with referring physicians. WES analysis was used to diagnose families suspected of having rare genetic disorders. The Genoox analysis platform was used for in-house bioinformatic analysis.

## Results

From 2014 to 2017, we studied 34 families. The most common referral reason was neurological manifestations (38%), with 50% of families being consanguineous. A definite diagnosis was achieved in 21 (61%) cases; 4 (11%) cases were diagnosed with variants in novel genes. Additionally, 6 (18%) families had strong candidate novel gene discoveries still under investigation. Therefore, the true diagnosis rate was probably higher. Some diagnoses were of significant impact, necessitating a change in patient management and provision of a tailored treatment.

## Conclusions

Accurate molecular diagnoses can pave the way for improved patient care and providing opportunities to study disease mechanisms, possibly leading to development of tailored treatments. Data from our genetic research program demonstrated high diagnostic and novel disease causative gene discovery rates. These high rates are most likely related to Northern Israel’s unique population, together with our case selection strategy, and close collaboration with analysts, geneticists, and clinicians, all within the same facility.

# Abstract #046. CO2 Laser Therapy for Female Stress Urinary Incontinence

**Published:** 2019-01

**Authors:** Roy Lauterbach, Hanin Dabajah, Emad Matanes, Ilan Gruenwald, Lior Lowenstein

**Affiliations:** 1 Division of Obstetrics and Gynecology, Rambam Health Care Campus, Haifa, Israel; 2 Neuro-Urology Unit, Rambam Health Care Campus, Haifa, Israel; 3 The Ruth & Bruce Rappaport Faculty of Medicine, Technion–Israel Institute of Technology, Haifa, Israel

## Background

Stress urinary incontinence (SUI) is of high prevalence among female patients causing an alarming effect on patients’ quality of life. Treatments of choice may include pelvic floor physiotherapy, targeted medication, or surgery. In recent years, a trend toward minimally invasive treatments has been noted. Laser-based devices have been recently introduced as an alternative minimally invasive treatment modality for SUI. Laser treatment has an ablative effect on the vaginal mucosa at first, followed by a rapid healing sequence including the formation of new collagen fibers and strengthening surrounding supporting tissue. On 30 July 2018, the US Food and Drug Administration (FDA) sent out a stern warning regarding the use of laser therapy for “vaginal rejuvenation” therapy aimed at treating symptoms of sexual dysfunction. Concerns were raised over potential short- and long-term adverse events.

## Study Aims

The aim of this study was to assess the efficacy and safety of a vaginal CO2-based laser device for treatment of female SUI.

## Materials & Methods

Inclusion criteria were 18–70-year-old female patients with clinically proven SUI. Exclusion criteria were prior suspicious PAP smear result, current urinary tract infections, vaginal bleeding, or pregnant state. Patients were requested to fill validated questionnaires regarding SUI and prolapse (UDI6 and ICIQ-UI). Three-month post-therapy follow-up was performed and assessed by a combination of physical examination, urodynamic testing, and questionnaire completion. Three sequential laser therapy sessions were performed without anesthesia. Each session lasted up to 5 minutes. Time interval between treatments was 1 month. The FDA-approved AcuPulse™ CO2 Laser system with an adapted vaginal probe (FemTouch™ handpiece by Lumenis, Yokneam Industrial Park, Israel) was utilized. Treatment was aimed at covering the entire vaginal mucosa area.

## Results

Of the 30 screened women, mean age 49.5, 24 women completed the 6-month participation period. Of these 24 women, 8.5% were post-menopausal. The average birth rate was 3.1. In 25% of cases, a low level of discomfort (stinging sensetion) that lasted up to 5 minutes was reported and had no effect on treatment completion. No adverse events occurred during or after treatment. Three months after the last treatment session, 66% reported a significant clinical improvement, with an additional 16% reporting complete resolution of symptoms. Two patients reported no change in baseline complaints. Significant improvement in questionnaire scores was documented: a 4.9-point increase in the ICIQ-UI incontinence questionnaire (*P*= 0.0014) and a 16.7-point increase in the UDI6 questionnaire (*P*=0.0023) were reported. Over 70% of patients continued to test positive for SUI on the urodynamics test, even though reporting a satisfying subjective improvement in symptoms.

## Conclusions

CO2-based laser therapy is safe and effective according to short-term follow-up, representing a new minimally invasive modality for the treatment of SUI in female patients.

# Abstract #049. Pathological Quantitative Clitoral Sensory Testing in Female Patients with Orgasmic Disorder

**Published:** 2019-01

**Authors:** Roy Lauterbach, Saar Aharoni, Lior Lowenstein, Ilan Gruenwald

**Affiliations:** 1 Division of Obstetrics and Gynecology, Rambam Health Care Campus, Haifa, Israel; 2 Neuro-Urology Unit, Rambam Health Care Campus, Haifa, Israel; 3 The Ruth & Bruce Rappaport Faculty of Medicine, Technion–Israel Institute of Technology, Haifa, Israel

## Background

Female sexual dysfunction (FSD) is a common multifactorial disorder affecting 30%–50% of sexually active women. It is defined as a disorder in sexual desire, orgasm, arousal, and pain during intercourse that results in significant personal distress.

## Study Aims

The objective of this study was to evaluate the association between orgasmic disorder in women in whom orgasmic disorder was their primary complaint and quantitative sensory testing deficiency.

## Materials & Methods

Patients with FSD were selected from the Sexual Dysfunction Clinic in the hospital. The study group comprised women in whom orgasmic dysfunction was their primary complaint. The control group was an age-adjusted group of women in whom orgasmic disorder was not their primary complaint. Sexual dysfunction was assessed by the female sexual function index (FSFI) questionnaire. Quantitative sensory testing was performed with a thermal and vibration Genito-Sensory Analyzer (GSA; Medoc Ltd., Israel) aimed at the clitoral and vaginal area.

## Results

Overall, 199 women with a mean age of 37.5±11.6 years were included in the study. As expected, FSFI orgasmic sub-domain scores were significantly higher in the study group compared to the control group (1.91±1.3 vs. 0.97±0.94, *P*<0.001, respectively). Total FSFI scores were also significantly higher in the study group compared with the control group (15.6±3.6 vs. 11.9±3.2, *P*<0.001). Our findings demonstrated significantly higher clitoral vibratory sensory thresholds in the study group compared with the control group (2.02 [1.12–2.64] vs. 1.55 [1.12–2.41], *P*<0.001, respectively).

## Conclusions

Women with orgasmic disorder as their main sexual disorder complaint proved to have other pathological sexual disorders in addition to their main complaint. GSA testing showed a possible correlation between vibratory clitoral stimulation and orgasmic disorder. Women with an orgasmic disorder as their main complaint required higher thresholds of clitoral stimulation. These findings may reinforce the belief in the role the clitoris plays in obtaining sexual orgasm, thus inferring a possible physiologic cause of orgasmic dysfunction beyond the proven psychological causes.

# Abstract #051. Penile Curvature—Conservative versus Surgical Treatment?

**Published:** 2019-01

**Authors:** Ilan Gruenwald, Raanan Tal, Boaz Appel

**Affiliations:** 1 Neuro-Urology Unit, Rambam Health Care Campus, Haifa, Israel; 2 The Ruth & Bruce Rappaport Faculty of Medicine, Technion–Israel Institute of Technology, Haifa, Israel

## Background

Penile deformity, painful erections, and erectile dysfunction are the major causes of anxiety and frustration in men with Peyronie’s disease (PD). In the past, the first-line therapy for PD was surgical repair. Recently, a new treatment modality was introduced—injection of collagenase, an enzyme derived from *Clostridium histolyticum*, directly into the penile plaque that causes the deformity.

## Study Aims

This study reviewed the considerations taken for offering the best individually matched type of therapy (injection of collagenase, surgery, or follow-up only) for PD patients, according to physician and patient preferences.

## Materials & Methods

From August 2015 to July 2018, retrospective data were collected on patients with varying levels of PD, including curvature size, plaque size and location, sexual function, patient preferences for therapeutic intervention, provided treatments, and complications.

## Results

During this period, 177 PD patients were identified. Fifty-three (30%) patients required no intervention due to minimal penile deviation and normal sexual function. Of the 124 who continued follow-up (mean 14 months), 74 were surgical candidates, and by the time the data were reviewed 46 (37%) had undergone surgery.

During follow-up we found that patients were concerned about penis shortening resulting from surgery, and expressed less concern about injection therapy. Only 18 were offered the option of injection therapy. Twenty-five were not suitable for injections due to the degree of curvature (3 patients with complex deformations, 5 with a significant lateral deviation, 4 with ventral curvature). Plaques were very solid (*n*=9) or inaccessible for injection (*n*=1). Two patients had severe erectile dysfunction, and 2 preferred optimal straightening of the penis. Of the remaining 18 that were suitable for both injection and surgery, the advantages/disadvantages of each method were explained; 15 chose injection therapy, and 3 chose surgery. A total of 15 (31%) had injections, the remaining 28 (63%) underwent surgical treatment, although the majority expressed concern about penile shortening. Three (6%) underwent prosthetic implantation. No treatment method had significant complications.

## Conclusions

Most PD patients are suitable candidates for surgery and curvature repair, while injection therapy is appropriate for a selected group of PD patients. The patient’s preference should be considered only after clarifying the advantages/disadvantages of each therapeutic approach. Injection therapy is attractive to patients who are concerned about penile shortening in surgery, but this treatment should be limited only to a select group of PD patients with higher chances of success.

# Abstract #052. PAX8 Plays an Essential Role in High-grade Serous Ovarian Carcinoma Via Activation of Mutant p53 and Mislocalized p21

**Published:** 2019-01

**Authors:** Dima Ghannam-Shahbari, Eyal Jacob, Reli Rachel Kakun, Tanya Wasserman, Lina Korsensky, Ofir Sternfeld, Juliana Kagan, Debora Rosa Bublik, Sarit Aviel-Ronen, Keren Levanon, Edmond Sabo, Sarit Larisch, Moshe Oren, Dov Hershkovitz, Ruth Perets

**Affiliations:** 1 The Clinical Research Institute at Rambam, Oncology Institute, Rambam Health Care Campus, Haifa, Israel; 2 The Ruth & Bruce Rappaport Faculty of Medicine, Technion–Israel Institute of Technology, Haifa, Israel; 3 Cell Death Research Laboratory, Department of Biology, Faculty of Sciences, University of Haifa, Haifa, Israel; 4 Department of Molecular Cell Biology, The Weizmann Institute of Science, Rehovot, Israel; 5 Department of Pathology, The Talpiot Medical Leadership Program, Sheba Medical Center, Ramat Gan, Israel; 6 Sheba Cancer Research Center, The Talpiot Medical Leadership Program, Sheba Medical Center, Ramat Gan, Israel; 7 Sackler Faculty of Medicine, Tel Aviv University, Ramat Aviv, Israel; 8 Department of Pathology, Rambam Health Care Campus, Haifa, Israel; 9 Department of Pathology, Tel Aviv Sourasky Medical Center, Tel Aviv-Yafo, Israel

## Background

The most common subtype of ovarian cancer is high-grade serous carcinoma (HGSC). Today it is widely accepted that this disease often originates from the fallopian tube epithelium. The fallopian tube lineage marker, PAX8, plays an essential role in development of the embryonal female genital tract. PAX8 is expressed in the adult fallopian tube secretory epithelial cells (FTSEC). Its expression is maintained through the process of FTSEC transformation to HGSC. Notably, in close to 100% of HGSCs, gain-of-function mutations occur in the tumor suppressor *TP53*, conferring p53 with oncogenic activities.

## Study Aims

The aim of this study was to delineate the role and mechanism of action of PAX8 in HGSC, and to study the connection between the ubiquitous PAX8 expression and p53 oncogenic activity in HGSC.

## Materials & Methods

Using RNA interference, we knocked down PAX8 expression in various HGSC cells and examined the effect on cell proliferation and expression levels of *TP53* and p21. Binding of PAX8 and p53 to specific regions in DNA was examined by chromatin immunoprecipitation followed by gene-specific qPCR. Immunofluorescence and immunohistochemistry were employed for examining p21 localization in normal and malignant tissues. Interaction of PAX8 with p53 in cells was studied using co-immunoprecipitation.

## Results

PAX8 was demonstrated to have a pro-proliferative and anti-apoptotic role in HGSC. In addition, it positively regulates *TP53* expression in HGSC, and the pro-proliferative role of PAX8 is mediated by the gain-of-function activity of mutant p53. Surprisingly, mutant p53 transcriptionally activates the expression of p21, which localizes to the cytoplasm of HGSC cells where it plays a non-canonical, pro-proliferative role. Furthermore, PAX8 and p53 interact in ovarian cancer cells and both bind to p21 promoter at p53 responsive element, suggesting that PAX8 and p53 act as transcriptional co-activators.

## Conclusions

We therefore propose a model by which the pro-proliferative role of PAX8 in HGSC is mediated by up-regulation of the mutated p53 and cytoplasmic p21. Mutated p53 and cytoplasmic p21 play a pro-proliferative role in HGSC, rather than their canonical tumor-suppressive role. We further suggest that PAX8 and p53 cooperate in HGSC to activate pro-proliferative target genes. Combined, these findings illustrate how a normal regulatory pathway is subverted by *TP53* mutations in HGSC into a driver of tumor progression.

# Abstract #053. A Novel Method to Estimate the Diameter of Abdominal Aortic Aneurysm

**Published:** 2019-01

**Authors:** Gil Zur, Maria Litvin, Eyal Berkovich, Maisa Andreus, Nira Beck, Diana Gaitini, Marcia Javitt

**Affiliations:** 1 Department of Medical Imaging, Rambam Health Care Campus, Haifa, Israel; 2 The Ruth & Bruce Rappaport Faculty of Medicine, Technion–Israel Institute of Technology, Haifa, Israel

## Background

Abdominal aneurysms larger than 5.5 cm are candidates for invasive treatment either with stent graft or aneurysmectomy. Currently, computed tomography angiography (CTA) or ultrasound (US) are used to estimate the size of the aneurysm. Radiation exposure and contrast injection are major drawbacks of using computed tomography (CT), while inaccurate measurements are known pitfalls of using US.

## Study Aims

To evaluate whether CT-US fusion is an accurate method to measure abdominal aneurysm and determine if it can replace current imaging techniques.

## Materials & Methods

The study included 11 patients with infra-renal aortic aneurysm and recent abdominal CTA. Each of the patients had a conventional US exam to estimate the aneurysm size, and immediately thereafter a CT-US fusion exam to measure the aneurysm again, this time based on registration to the recent CTA. Gold standard values for aneurysm size were extracted from the CTA using PORTAL-PHILIPS, and marks of the maximal diameter were added to the DICOM files using MATLAB. The measurements of the fusion and conventional ultrasound were compared to the CTA values.

## Results

Fusion measurements were more accurate and reliable than the standard examination. Specifically it seems that good registration between the CTA and the US allowed for better estimation of fusiform aneurysms than using US alone.

## Conclusions

Our results suggest that CT-US fusion may prove to be a better study for follow-up of small abdominal aortic aneurysms. A larger cohort is needed to validate this result and to check whether the fusion technique can replace the CTA for follow-up of larger aneurysms, hence reducing exposure to radiation and contrast material.

# Abstract #055. The Correlation between PIGU Expression and Radioactive Iodine Resistance in Patients with Well Differentiated Thyroid Cancer

**Published:** 2019-01

**Authors:** Shorook Na’ara, Moran Amit, Salem Billan, Shadi Shinnawi, Ziv Gil

**Affiliations:** 1 Laboratory for Applied Cancer Research, Rambam Health Care Campus, Haifa, Israel; 2 Department of Otolaryngology-Head & Neck Surgery, Head and Neck Center, Rambam Health Care Campus, Haifa, Israel

## Background

Differentiated thyroid cancer (DTC) accounts for the vast majority of thyroid cancers. In cases of locally advanced disease or loco-regional recurrence after surgical treatment, radioactive iodine (RAI) is administered. However, patients vary in their response to RAI treatment. The endoplasmic reticulum protein, PIGU, is a part of the GPIT complex.

## Study Aims

This study examined the post-translational effect of PIGU on the sodium/iodide symporter (NIS) in DTC, and the correlation between PIGU expression in cancerous tissue and responsiveness to RAI treatment.

## Materials & Methods

We analyzed the level of PIGU expression in normal thyroid tissue and in papillary thyroid carcinoma (PTC) tissue in freshly excised patient specimens. Next, we performed *in vitro* studies using DTC human cell line (K1) and DTC cells overexpressing PIGU (K1-PIGU), both generated at our lab by stable transfection. Mice were injected orthotopically with K1 or K1-PIGU cells and subjected to RAI treatment followed by PET scan to evaluate iodine uptake.

## Results

PIGU was significantly under-expressed in the tumor compared to the matched normal thyroid tissue (*P*<0.001). Expression of PIGU in human papillary carcinoma cells increased sodium iodide symporter expression and subsequently RAI uptake *in vitro* and *in vivo*. Furthermore, we found that the mitogen-activated protein kinase (MAPK) inhibitor U0126 can alter PIGU expression. Finally, positive staining for PIGU in the primary tumor was associated with disease-free survival (*P*<0.01) and distant metastasis-free survival (*P*<0.01) after treatment with RAI. We found that PIGU expression affects disease progression. Negative staining for PIGU resulted in more advanced disease with distant metastasis, which worsened survival. Multivariate analysis showed that PIGU expression, age, and gender were independent factors for DFS.

## Conclusions

Our findings represent a novel mechanism for RAI resistance. PIGU expression in DTC is correlated with a better response to RAI treatment.

# Abstract #056. Paracrine Interactions between Schwann Cells and Cancer Cells Promote Perineural Invasion Via L1CAM Secretion

**Published:** 2019-01

**Authors:** Shorook Na’ara, Moran Amir, Ziv Gil

**Affiliations:** 1 Laboratory for Applied Cancer Research, Rambam Health Care Campus, Haifa, Israel; 2 Department of Otolaryngology Head and Neck Surgery, the Head and Neck Center, Rambam Health Care Campus, Haifa, Israel

## Background

Cancerous neural invasion (CNI) is a well-known route of cancer spreading in malignant diseases, especially in cancers of the head/neck, prostate, and pancreatic cancer. Patients suffer from severe morbidity due to neuropathic pain induced by CNI, and most succumb to cancer within 1 year. The exact mechanism that drives cancer cells to disseminate along nerves is unknown. L1 cell adhesion molecule (L1CAM) is a transmembrane adhesion molecule responsible for nervous system development. However, it was found to be upregulated in many human cancers, and its expression is generally associated with poor prognosis and metastasis formation.

## Study Aims

Our study aim was to evaluate the mechanism by which L1CAM induce CNI in pancreatic cancer.

## Materials & Methods

Immunohistochemical analysis of the neural niche was carried out in cancer specimens excised from humans for expression of L1CAM and markers of cellular components of the neural niche. A novel *in vitro* nerve invasion assay that consists of three layers of fibroblasts–extracellular matrix–Schwann cells (SC) was used to assess cancer cell invasion through nerves *in vitro*, and the role of L1CAM secreted from SC in the process. Transgenic KPC mice were used for *in vivo* studies.

## Results

First, we analyzed the expression pattern of L1CAM in pancreatic cancer specimens and found that L1CAM is overexpressed in the areas of invaded nerves in cancer cells and in Schwann cells. L1CAM secreted from SC enhanced the migration and motility of the cancer cells shown by migration and invasion assays and acted as chemo-attractant to cancer cell movement. By the modified three-layer *in vitro* invasion assay we showed that L1CAM knock-down in Schwann cells impaired the cancer cell invasion through these layers. Furthermore, we found that L1CAM secreted from SC specifically induced MAPK signaling pathway activation through Erk phosphorylation. L1CAM also upregulated expression of matrix metalloproteinase-2 (MMP-2) and MMP-9 by PDAC cells, through STAT3 phosphorylation. Finally, *in vivo* studies in transgenic mice treated with L1CAM monoclonal antibody by intraperitoneal injection showed reduced CNI in the tumors compared to the control group.

## Conclusions

We describe a novel mechanism that promotes cancer neural invasion in pancreatic cancer via L1CAM secretion and paracrine effect between Schwann cells and the cancer cells.

# Abstract #057. New Outpatient Flowmeter Device for Monitoring Male Voiding Dysfunction

**Published:** 2019-01

**Authors:** Ilan Gruenwald, Ravit Yehieli-Cohen, Omar Masarwa, Boaz Appel

**Affiliations:** 1 Neuro-Urology Unit, Rambam Health Care Campus, Haifa, Israel; 2 The Ruth & Bruce Rappaport Faculty of Medicine, Technion–Israel Institute of Technology, Haifa, Israel

## Background

Uroflowmetry is an important test for evaluating voiding difficulties, which is performed in a clinical set-up. The disadvantages of such a study are that it is performed during a single episode of micturition, and does not necessarily represent the true status of daily micturitions. Results may also be affected by the clinically unnatural environment.

The PeePal system (P. Square Medical Ltd, Herzliya, Israel) is a new device recently developed for obtaining uroflow parameters at home, in a repetitive manner to enhance reliability and statistics.

## Study Aims

This study compared uroflow parameters measured by a standard clinic uroflowmeter device with the PeePal system and evaluated its feasibility as a portable device for testing in any natural environment.

## Materials & Methods

Males aged 18–85 who were invited for a urodynamic test and gave consent used the PeePal system during uroflowmetry. This was possible because the device comprises a portable recorder and a non-invasive vibro-acoustic, plastic ring-like sensor, which is placed on the dorsum of the penis and senses the urethral vibration during urination. The recorded signal is analyzed offline to provide a uroflow curve and parameters. The results were compared to standard uroflowmetry test for statistical and correlation evaluation.

## Results

Both tests were measured simultaneously in 48 male patients. The quantitative measures chosen for comparison in both tests were maximum flow (Qmax) and voided volume (VV). There was no significant statistical difference between the two methods, demonstrating the similarity of both test results.

Average Qmax on the uroflow device was 13.7± 6.1 mL/s compared to 15.2±9.52 mL/s for the PeePal system. Average VV on the uroflow device was 291.6±190.5 mL compared to 312±193 mL for the PeePal system, indicating a good correlation (*P*= 0.22, *P*=0.29, respectively).

The PeePal system showed overall good correlation to the standard uroflowmetry test. It was also found to be applicable and convenient. The wearable sensor is easily self-mounted, does not cause any inconvenience to the patient, and does not interrupt normal urination.

## Conclusions

The quantitative measures showed excellent correlation between the two devices, making the PeePal system a promising new technology for repetitive convenient measures of urine flow throughout the day in a home/work/natural environment. This study is now being extended to evaluate the device to provide data on obstructive vs. non-obstructive flow patterns, in place of standard pressure flow studies that require patient catheterization.

# Abstract #058. The Selective Uptake of Exosomes Secreted by Macrophages into Pancreatic Ductal Adenocarcinoma

**Published:** 2019-01

**Authors:** Lana Ginini, Neta Milman, Ziv Gil

**Affiliations:** 1 Laboratory for Applied Cancer Research, The Clinical Research Institute at Rambam, Rambam Health Care Campus, Haifa, Israel; 2 Department of Otolaryngology Head & Neck Surgery, Head and Neck Center, Rambam Health Care Campus, Haifa, Israel

## Background

Pancreatic ductal adenocarcinoma (PDAC) is a leading cause of cancer-related death, with low survival rates. Tumor-associated macrophages (TAM), a cardinal component of tumor mass, have a key role in tumor progression and metastasis. Recently, we and others have shown that cells can communicate through exosomes in addition to secretion of soluble factors and cell–cell adhesion.

## Study Aims

The aim of the research was to discover the fundamental mechanism of macrophage-derived exosomes’ (MDEs) interaction with PDAC cells and decipher their cell specificity.

## Materials & Methods

Exosomes were purified from polarized peritoneal macrophage-conditioned media by a series of ultracentrifugation. Isolated exosomes were stained with carboxyfluorescein diacetate succinimidyl-ester (CFSE). To investigate the internalization process of MDEs by microscopy and flow cytometry, primary culture of PDAC and fibroblast cells were seeded in the presence/absence of CFSE-labeled exosomes. For uptake inhibition assays PDAC cells were pre-treated with various pharmacological inhibitors.

## Results

Co-culture of fluorescently labeled macrophages and PDAC cells in the presence of GW4869, an exosome biogenesis inhibitor, resulted in marked blocking of exosome transfer from macrophages to PDAC cells. Following direct addition of MDE to PDAC cells, CFSE exosomes were observed entering the PDAC cells and remained in the PDAC cytoplasm rather than attaching to the cell surface. The MDEs applied to primary cultures of PDAC cells and fibroblasts showed labeled exosome localization inside PDAC cells, with no apparent internalization into fibroblasts. Furthermore, we demonstrated a role for proteins on the exosome surface for uptake, since exosomes treated with proteinase-K showed a reduction of nearly 50% in uptake compared to the non-treated group.

Flow cytometry results showed that PDAC cells treated with nocodazole, an endocytosis inhibitor, had a significantly lower percentage of CFSE-positive cells compared to the non-treated group. However, treating PDAC cells with 5-(N-Ethyl-N-isopropyl) amiloride (EIPA; 25 μM) and pitstop (15 μM) inhibitors for micropinocytosis and clathrin-mediated endocytosis, respectively, did not inhibit CFSE exosome accumulation into PDAC cells.

## Conclusions

We obtained a comprehensive picture and confirmed that MDEs are readily and preferentially internalized by PDAC cells, utilizing proteins expressed on their surface to enter the cells. The data also suggest that inhibition of endocytosis disrupts the ability of cancer cells to internalize MDEs. However, MDE endocytosis is not mediated by micropinocytosis and clathrin-mediated endocytosis.

# Abstract #059. The Relationship between the Quality of New Medication (Insulin or Anti-coagulation Therapy) Counseling and Patients’ Perceptions of Their Transitional Care

**Published:** 2019-01

**Authors:** Khalil Namora

**Affiliations:** Department of Internal Medicine B, Rambam Health Care Campus, Haifa, Israel

## Background

With the increase in life expectancy and comorbidity, reduced hospitalization days, and the fragmented structure of the Israeli health system, patient discharge requires optimal planning.

Patient counseling toward transfer to the community is important, particularly when discharging a patient with a new medical treatment.

## Study Aims

The aim of this study was to examine the transition quality from hospital to community care, in patients discharged with a new insulin or anticoagulant treatment. Secondary aims were to identify patient knowledge gaps and to assess the relationship between new medication patient counseling and the patients’ levels of understanding and readiness for self-care.

## Materials & Methods

This was a cross-sectional study that included patients discharged from various inpatient departments, and who agreed to complete questionnaires at the time of discharge. Patients were surveyed on their socio-demographic characteristics, the quality of counseling given at the time of discharge, and their levels of understanding the instructions. Additionally, patients were asked to complete the Care Transition Measure (CTM), a validated measure of patient experiences with the quality of their transitional care.

Statistical analysis included descriptive statistics for socio-demographic variables and the study variables, as well as analysis of the relationship between the quality of the new medication counseling given, patients’ understanding of their new medication treatment regimen, and CTM ratings.

## Results

This study included 79 patients, 56% of whom were men; mean age 61 years. A non-significant tendency was found (*P*=0.06) for an inverse relationship between socioeconomic status and the care transition experience, such that the higher the socioeconomic status, the lower the transitional care ratings. A total of 41 (52%) patients assessed their transitional care experience as very good (CTM score 85 or higher). In uni-variate analysis, all the counseling and understanding items (except for explanations on “duration of medication regimen”) were significantly associated with high CTM scores. The only counseling item that remained significantly related to high CTM scores in the multivariate analysis was “understanding the new drug therapy that had been added during the hospital stay.”

## Conclusions

The study showed that addressing new treatments added during their hospital stay as part of the discharge briefing process was clearly related to a high degree of preparedness and continued self-treatment. Thus, the study adds to the understanding of the importance of pre-discharge instruction to patients placed on a new drug therapy.

# Abstract #060. A Novel Homozygous Missense Mutation in TUFM Leads to a Mitochondrial Cardiomyopathy Syndrome without Progressive Encephalopathy

**Published:** 2019-01

**Authors:** Tova Hershkovitz, Alina Kurolap, Tamar Paperna, Adi Mory, Regeneration Genetics Center, Hanna Mandel, Hagit Baris Feldman

**Affiliations:** 1 The Genetics Institute, Rambam Health Care Campus, Haifa, Israel; 2 The Ruth & Bruce Rappaport Faculty of Medicine, Technion–Israel Institute of Technology, Haifa, Israel; 3 Regeneron Genetics Center, Tarrytown, New York, USA; 4 Institute of Human Genetics and Metabolic Disorders, Western Galilee Medical Center, Nahariya, Israel

## Background

Mitochondrial dysfunction typically leads to a spectrum of clinically heterogeneous, and mostly devastating, disorders. Accurate molecular diagnosis of mitochondrial disorders is often difficult, posing significant challenges in clinical decision making and genetic counseling. We describe a proband who was the third son of healthy consanguineous parents of Muslim Arab descent. He presented at 6 months with severe cardiogenic shock and lactic acidosis. Multiple mitochondrial respiratory complex dysfunctions were observed on muscle biopsy. He died within 1 month.

## Study Aims

The aim of this study was to provide a genetic diagnosis for the family upon the early demise of their third, previously healthy, child.

## Materials & Methods

We performed trio whole-exome sequencing (WES) for the patient and parents in collaboration with the Regeneron Genetics Center. Given the phenotype presentation and reported consanguinity of the parents, segregating variants were further analyzed for rare protein-altering and recessively inherited variants in genes related to mitochondrial function. Validation of the candidate variant and segregation analysis were done by Sanger sequencing. Protein structures were modeled using SWISS-MODEL based on the bovine crystal structure of mitochondrial Elongation Factor Tu/Ts complex (PDB #1XB2), and visualized with UCSF Chimera software.

## Results

We identified a novel homozygous missense variant in TUFM (NM_003321.4): c.344A>C; p.His115Pro, encoding the mtDNA translation elongating factor Tu. Histidine at position 115 is located in domain 1 of the EFTu protein, which is required for GTP/GDP-binding and for complex formation with elongation factor Ts (EFTs). The highly conserved His115 (GERP 5.42) is predicted to be an exposed and functional residue; therefore, the substitution of histidine with a physiochemically different proline may compromise proper protein folding and disrupt the function of this domain. Based on EFTu protein structure and the location of His115, the His115Pro substitution may hamper Tu/Ts complex stability and EFTu reactivation.

## Conclusions

Mutations in genes involved in mtDNA translation, such as TUFM, are increasingly implicated in severe mitochondrial disease. To date, only four patients have been reported with bi-allelic TUFM mutations, causing combined oxidative phosphorylation deficiency 4 (COXPD4) with severe early-onset lactic acidosis and progressive fatal infantile encephalopathy. The patient presented here expands the phenotypic features of TUFM-related mitochondrial disease, as he presented with lactic acidosis and dilated cardiomyopathy without early encephalopathy. Our findings warrant the inclusion of TUFM in differential diagnosis of early-onset cardiomyopathy with suspected mitochondrial etiology.
